# Evaluation of CRISPR Diversity in the Human Skin Microbiome for Personal Identification

**DOI:** 10.1128/mSystems.01255-20

**Published:** 2021-02-02

**Authors:** Kochi Toyomane, Ryo Yokota, Ken Watanabe, Tomoko Akutsu, Ai Asahi, Satoshi Kubota

**Affiliations:** a National Research Institute of Police Science, Kashiwa, Chiba, Japan; University of Trento

**Keywords:** human skin microbiome, CRISPR, metagenomics, forensic science, next-generation sequencing

## Abstract

The highly personalized human skin microbiome may serve as a viable marker in personal identification. Amplicon sequencing resolution using 16S rRNA cannot identify bacterial communities sufficiently to discriminate between individuals. Thus, novel higher-resolution genetic markers are required for forensic purposes. The clustered regularly interspaced short palindromic repeats (CRISPRs) are prokaryotic genetic elements that can provide a history of infections encountered by the bacteria. The sequencing of CRISPR spacers may provide phylogenetic information with higher resolution than other markers. However, using spacer sequencing for discrimination of personal skin microbiome is difficult due to limited information on CRISPRs in human skin microbiomes. It remains unclear whether personal microbiome discrimination can be achieved using spacer diversity or which CRISPRs will be forensically relevant. We identified common CRISPRs in the human skin microbiome via metagenomic reconstruction and used amplicon sequencing for deep sequencing of spacers. We successfully reconstructed 24 putative CRISPR arrays using metagenomic data sets. A total of 1,223,462 reads from three CRISPR arrays revealed that spacers in the skin microbiome were highly personalized, and conserved repeats were commonly shared between individuals. These individual specificities observed using CRISPR typing were confirmed by comparing the CRISPR diversity to microbiome diversity assessed using 16S rRNA amplicon sequencing. CRISPR typing achieved 95.2% accuracy in personal classification, whereas 16S rRNA sequencing only achieved 52.6%. These results suggest that sequencing CRISPRs in the skin microbiome may be a more powerful approach for personal identification and ecological studies compared to conventional 16S rRNA sequencing.

**IMPORTANCE** Microbial community diversity analysis can be utilized to characterize the personal microbiome that varies between individuals. CRISPR sequences, which reflect virome structure, in the human skin environment may be highly personalized similar to the structures of individual viromes. In this study, we identified 24 putative CRISPR arrays using a shotgun metagenome data set of the human skin microbiome. The findings of this study expand our understanding of the nature of CRISPRs by identifying novel CRISPR candidates. We developed a method to efficiently determine the diversity of three CRISPR arrays. Our analysis revealed that the CRISPR spacer diversity in the human skin microbiome is highly personalized compared with the microbiome diversity assessed by 16S rRNA sequencing, providing a new perspective on the study of the skin microbiome.

## Abstract (precis)

Microbial community diversity analysis can be utilized to characterize the personal microbiome that varies between individuals. CRISPR sequences, which reflect virome structure, in the human skin environment may be highly personalized similar to the structures of individual viromes.

## INTRODUCTION

Recent studies have revealed significant variation in the structure of the human skin microbiome among healthy individuals (1–3). Fierer et al. demonstrated that bacterial communities on the hands of different people share only 13% identity (4). Several studies have demonstrated individuality of the microbiome as a “bacterial fingerprint” to develop a novel method for the identification of people involved in crime scenes, in which metagenome data sets or 16S rRNA sequencing data were analyzed (1, 5, 6). It is expected that bacterial DNA can be easily recovered from the surface of objects touched by a suspect, considering the abundance of bacteria on human skin (1). The microbiome of human skin has gained attention in recent years owing to low success rates of DNA profiling from an object touched by hands, which typically yields a small amount of DNA (7). Identification of individuals in crime scenes based on microbial community diversity requires characterization of the microbiome community with a degree of high resolution. Schmedes et al. utilized whole-genome shotgun sequencing to discriminate personal microbiomes using a strain-level taxonomic profiling (5). Although the study achieved high accuracy for personal identification by analyzing public data, the high cost and complexity of analysis precludes its practical use. In contrast, amplicon-based microbiome profiling is comparatively cost-effective, as it can be performed using conventional methods such as microarray analysis (8–11). As a phylogenetic marker, the 16S rRNA gene has been widely used in microbiome studies. However, 16S rRNA-based personal identification is easily perturbed over time (12, 13). The temporal instability of the “bacterial fingerprint” is likely due to loss of minor species in an environment. Moreover, the resolution of amplicon sequencing using 16S rRNA is not sufficient to discriminate bacteria at the strain level or, in many cases, at the species level (14). The importance of strain-level classification of specific species for personal identification was previously proposed (5, 15). Effective personal identification would require determination of the microbial community composition at high resolution; thus, novel strategies are needed to employ amplicon sequencing for forensic purposes.

The clustered regularly interspaced short palindromic repeat (CRISPR) array corresponds to a genomic locus found in prokaryotes and is composed of identical repeats, formerly called direct repeats, and unique sequences between repeats, called spacers (16). Typically, CRISPR repeats range from 28 to 37 bp in length, whereas spacers range between 32 and 38 bp (17). Each CRISPR corresponds roughly to a specific taxon. However, some strains harbor multiple CRISPR loci and some CRISPRs are conserved in multiple species. CRISPRs, along with CRISPR-associated proteins (Cas), comprise the adaptive immune system of prokaryotes (18). It serves as a defensive mechanism against foreign genetic entities, such as phages. To provide immunity against the reinfecting foreign DNA, the CRISPR-Cas system integrates a short DNA fragment derived from the foreign DNA as a new spacer. Therefore, spacer sequences in a CRISPR array represent a history of past infection encountered by a bacterial strain and reflect the structure of the viral community, which is more personalized than that of the bacterial community itself (19, 20).

As variable spacers in a CRISPR array are flanked by conserved repeats, it can be used as a phylogenetic marker that provides higher resolution than other markers, such as 16S rRNA. CRISPR typing using the spacer acquisition history of CRISPR arrays has been utilized for phylogenetic analysis of bacteria (21–30, and see reference 31 for a review). This typing strategy was also applied in some metagenomic studies to characterize complex microbial populations or identify host-virus interactions, both by reconstruction of CRISPRs from shotgun metagenomic data sets (32–35) and amplicon sequencing utilizing primers targeting the repeats of CRISPR (20, 36–38). A limitation of CRISPR typing for microbiome characterization is that the spacers of a specific CRISPR locus recovered from a shotgun metagenome data set may be insufficient to understand spacer diversity within the microbial community. For example, Rho et al. could not identify the distribution of CRISPR arrays in some skin data sets by CRISPR array reconstruction using shotgun metagenome data sets obtained from the Human Microbiome Project (33). Another limitation is that amplicon sequencing targeting CRISPR arrays is strongly dependent on *a priori* knowledge of repeat sequences (32); CRISPR spacers cannot be sequenced by amplicon sequencing without understanding which CRISPR arrays are in a specific environment, whereas amplicon sequencing can be utilized for complete sequencing of spacers in a specific CRISPR locus. Therefore, it remains unclear whether it is possible to discriminate personal microbiomes using spacer diversity or identify the CRISPRs that are suitable for personal identification. To overcome these limitations, the identity of CRISPRs that are common in the human skin microbiome must be determined to enable characterization of the spacer diversity of CRISPRs in different individuals.

Here, we combined metagenomic and amplicon sequencing to overcome the limited information on CRISPR loci in the skin microbiome. We first identified the most abundant CRISPR repeats in the human skin microbiome via metagenomic reconstruction of CRISPR arrays. We then sequenced the spacers of three putative CRISPR arrays in the skin microbiome to characterize the diversity of spacer sequences and evaluate the potential of CRISPR array sequencing as a novel tool in personal identification. We validated our CRISPR-based analysis by comparing the pipeline with conventional 16S rRNA sequencing.

## RESULTS

### Identification of conserved CRISPR arrays in skin microbiomes among individuals by using metagenome data sets.

Amplicon-based CRISPR sequencing cannot be used without prior knowledge of the CRISPRs present in a microbial community. Thus, we first investigated the diversity of CRISPRs in metagenome data sets of skin samples obtained in a previous study (2) via metagenomic reconstruction of CRISPRs. Although reconstructions of the CRISPR arrays in the human microbiome were previously reported (33, 34), the diversity of CRISPR arrays in the skin metagenomic data remained unclear. Here, we focused on the data of samples collected from the hypothenar palm (Hp) region, as DNA from the hands are most likely to be collected from crime scenes. The metagenomic data were assembled using the *de novo* assembler MEGAHIT (39). CRISPR arrays were identified by PILER-CR, a tool developed for identifying CRISPRs (40). Using this pipeline, we identified 862 arrays from 15 samples among 14 individuals (see Data Set S1, sheet 1, in the supplemental material). No CRISPR arrays were obtained from samples MET0022 (HV01), MET0161 (HV08), or MET0177 (HV07). This may be attributed to insufficient assembly, as the number of reconstructed CRISPR arrays greatly depends on the assembly quality. Interestingly, two samples, MET0177 (HV07; Hp-L) and MET0190 (HV07; Hp-R) derived from the same individual, yielded metagenomes with different detectable CRISPR arrays. These results suggest that the repeatability of metagenomic reconstruction is insufficient to fully measure CRISPR diversity.

10.1128/mSystems.01255-20.7 DATA SET S1 (Sheet 1) Summary of metagenomic CRISPR reconstruction using MEGAHIT and PILER-CR. (Sheet 2) DR sequences commonly detected in skin metagenome datasets. (Sheet 3) Summary of CRISPRCasFinder results. (Sheet 4) Numbers of reads containing the CRISPR repeat in the samples. (Sheet 5) Sequencing statistics for spacer sequencing. (Sheet 6) Results of a BLAST search of S. equinus spacers. (Sheet 7) Results of a BLAST search of S. thermophilus spacers. (Sheet 8) Results of a BLAST search of M. luteus spacers. (Sheet 9) Metadata for all samples collected in this study. (Sheet 10) List of *Streptococcus* strains used as reference CRISPRs. (Sheet 11) Primer sequences used in this study. Download Data Set S1, XLSX file, 0.1 MB. Copyright © 2021 Toyomane et al. 2021 Toyomane et al. This content is distributed under the terms of the Creative Commons Attribution 4.0 International license.

**FIG 1:**
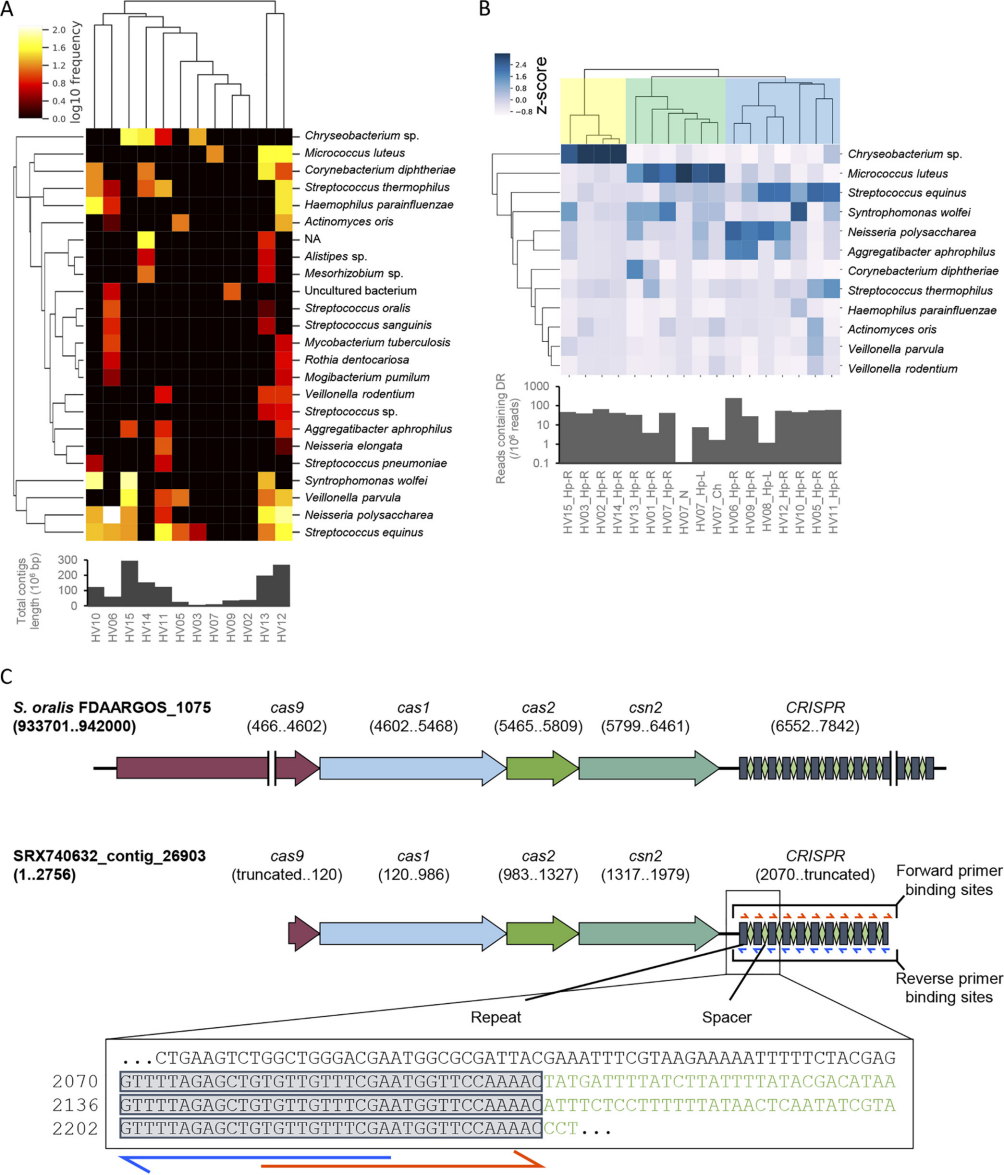
Metagenomic reconstruction of CRISPR arrays identified commonly shared repeats in skin metagenome data sets. (A) Heat map of the repeat matrix depicting the abundance of each repeat derived from putative CRISPR arrays detected in metagenomic data of the bacterial community on human skin. The bar chart below the heat map shows the total contig length after *de novo* assembly. (B) Heat map indicating the abundance of CRISPR repeats in metagenomic data sets detected by BLAST search against SRA data. Each column was standardized by the Z-score. The bar chart below the heat map shows reads containing repeats used as queries in the BLAST search. Hp, palm; N, nares; Ch, cheek. (C) Representative putative S. equinus CRISPR-Cas system detected by metagenomic reconstruction. CRISPR-Cas locus of Streptococcus oralis strain FDAARGOS_367 (GenBank CP023507) is shown as the reference. The presence of the characteristic *csn2* gene in the locus suggests that this system is type II-A CRISPR-Cas system; the *cas9* gene, the effector protein of type II system, was partially detected in the reconstructed CRISPR array, because the 5′ end of the entire CRISPR-Cas locus was truncated. The box at the bottom indicates the partial sequence of the CRISPR array. The marked sequences are repeat sequences, while the sequences colored in green are spacer sequences. Forward and reverse primer binding sites on the repeats are indicated by red and blue arrows, respectively. The full sequence of the contig is provided in Text S1 in the supplemental material.

The origins of the repeats identified via metagenomic CRISPR reconstruction were identified by a BLAST search. Among 24 CRISPR repeat sequences, 14 CRISPR repeats were identical to their respective reference genomes, while the other 10 did not match exactly to the reference genomes (Data Set S1, sheet 2). Thus, the latter repeats may have been derived from previously unknown CRISPR arrays, although it remains unknown whether these putative CRISPRs were correctly reconstructed. Note that, in such cases, the annotation of CRISPRs, including host species, subtyping, or orientation, might not be precise or be impossible and should be clarified in future studies. The most frequently observed repeat was derived from Streptococcus equinus, which was observed in eight individuals (Fig. 1A; Data Set S1, sheet 1). This specific streptococcal repeat has been targeted in other studies using specific primers to amplify the spacers of *Streptococcus* species in the oral and skin microbiomes (38). In total, 24 putative CRISPR arrays were observed for at least two individuals, indicating that some CRISPR arrays were conserved among the population. To validate the reconstructed CRISPR arrays, we subtyped the CRISPR-Cas system based on nomenclature proposed by Makarova et al. (41) using CRISPRCasFinder (42) and compared the arrays with a known CRISPR database if *cas* genes were also detectable. Using CRISPRCasFinder, 42 CRISPRs that associated with *cas* genes were identified (Data Set S1, sheet 3); a representative CRISPR-Cas locus is shown in Fig. 1C. We compared some examples of CRISPR arrays with the most relevant arrays in a public database. For the most abundant CRISPR, S. equinus, we identified three CRISPR arrays by CRISPRCasFinder, one of which was subjected to a BLAST search to identify the most relevant CRISPR loci in the NCBI database. We found that the most similar CRISPR-Cas region for S. equinus CRISPR (see Fig. S1A) was that of Streptococcus oralis strain FDAARGOS_367 (GenBank CP023507), and S. equinus CRISPR was classified into type IIA, which is characterized by the *cas9* gene (Fig. S1B). Another example of CRISPR loci with *cas* genes was that of Streptococcus thermophilus. For S. thermophilus CRISPR, we obtained one contig containing both CRISPR and *cas* genes (Fig. S1C) that was close to the CRISPR-Cas system of Streptococcus mitis strain S022-V3-A4 (GenBank CP047883), which was also classified into type IIA (Fig. S1D). Repeats of both S. equinus CRISPR and S. thermophilus CRISPR were identical to those of CRISPR1 and CRISPR3 of Streptococcus thermophilus (43), although the entire loci were close to those of other *Streptococcal* species, suggesting that these CRISPRs are widely preserved among *Streptococcus* organisms. We then compared three representative S. equinus CRISPRs, which were detected with *cas* genes, against those with the same repeats (see Fig. S2). Given that all spacers in the reconstructed arrays were unique among the 25 references in CRISPRCasdb (44), it is difficult to precisely identify the array origin. Expansion of the CRISPR database may improve classification accuracy in the future. Altogether, the results suggest that CRISPR reconstruction using metagenomic data accurately detects repeats present in the skin microbiome.

10.1128/mSystems.01255-20.1 FIG S1 Summary of the CRISPR locus structure analyzed by CRISPRCasFinder. The loci structure of S. equinus CRISPR (A), Streptococcus oralis strain FDAARGOS_367-derived CRISPR (B), S. thermophilus CRISPR (C), and Streptococcus mitis strain S022-V3-A4-derived CRISPR (D). Download FIG S1, PDF file, 1.8 MB. Copyright © 2021 Toyomane et al. 2021 Toyomane et al. This content is distributed under the terms of the Creative Commons Attribution 4.0 International license.

10.1128/mSystems.01255-20.2 FIG S2 Alignment of the representative metagenomically reconstructed streptococcal CRISPR arrays against reference CRISPRs. Spacers of three S. equinus CRISPRs detected by CRISPRCasFinder (42) and 26 streptococcal reference CRISPRs were visualized by CRISPR Visualizer (71). Each colored box represents a spacer. Gray boxes with “x” represent missing spacers. Note that similar colors do not indicate similar sequences. All metagenomically reconstructed spacers are unique among the 26 arrays. Download FIG S2, PDF file, 0.1 MB. Copyright © 2021 Toyomane et al. 2021 Toyomane et al. This content is distributed under the terms of the Creative Commons Attribution 4.0 International license.

10.1128/mSystems.01255-20.8 TEXT S1 FASTA formatted sequence of the contig containing the CRISPR loci shown in Fig. 1C. Download Text S1, TXT file, 0.1 MB. Copyright © 2021 Toyomane et al. 2021 Toyomane et al. This content is distributed under the terms of the Creative Commons Attribution 4.0 International license.

### CRISPR repeat abundance in the skin microbiome.

Insufficient CRISPR arrays were detected in multiple data sets, with some individuals sharing no CRISPR arrays with others, perhaps due to the low coverage of CRISPR arrays. Reads containing CRISPR repeats may be observed more frequently in metagenome data sets, even in those where no CRISPR arrays were reconstructed. For repeats observed in metagenomic CRISPR reconstruction, we measured the number of reads containing each repeat using BLAST. We enumerated the reads containing repeats by searching the repeat sequence as a query against the metagenome data set using the Sequence Read Archive as a subject. Repeats observed in at least three individuals in the CRISPR reconstructions were included as queries for read counting. Among the CRISPR repeats identified in CRISPR reconstructions, the repeats derived from S. equinus, S. thermophilus, Neisseria polysaccharea, and Syntrophomonas wolfei were most frequently observed in read counting (Fig. 1B; Data Set S1, sheet 4, repeat identifiers are listed in sheet 2). Reads containing the repeats of these CRISPRs were detected from the hands of 14 of the 15 individuals by read counting, while those CRISPRs were observed in 8, 5, 6, and 3 individuals, respectively, by metagenomic CRISPR reconstructions (Data Set S1, sheet 2). As shown in Fig. 1B, individuals were categorized into three clusters based on the most abundant repeat; the most abundant repeats in the first and the second groups were derived from Micrococcus luteus and *Chryseobacterium*, respectively. In the last group, repeats derived from M. luteus and *Chryseobacterium* were less abundant than other repeats, such as those derived from S. equinus or N. polysaccharea. Clustering of repeat abundances was plausible because four samples from the same individual (HV07), including two samples each from nares and cheek (representing moist and oily sites, respectively), were grouped into the same cluster. Some repeats were observed in individuals even when no CRISPR array was detected in the assembled contigs (HV01 and HV08), suggesting that the prevalence of CRISPR arrays was underestimated by metagenomic reconstruction. Therefore, amplicon sequencing may be a more effective tool for studying CRISPR diversity.

### Stability of evenness and richness of CRISPR spacer sequences in the skin microbiome.

By reconstructing CRISPR arrays using metagenomic data, we detected putative CRISPR repeats commonly found in the skin microbiome. To test whether repeats detected in the reconstructed CRISPR arrays are also detected via PCR-based methods, we designed three pairs of primers that flank the spacer and target the repeats of S. equinus, S. thermophilus, and M. luteus, as they were frequently found during metagenomic reconstruction and are members of major genera on human skin (Fig. 1C). CRISPR spacers were amplified with these primer pairs and sequenced using MiSeq. Primers targeting the repeats were used rather than primers flanking the entire CRISPR locus to amplify a broader range of CRISPR arrays (36). Note that the spacer amplicon is a mixture of spacers derived from multiple species, since the same repeats are shared by several species. Thus, this approach cannot precisely determine which species is the origin of each spacer. Cutaneous skin swabs were collected from five individuals in their 20s to 40s and from the skin sites Hp, antecubital fossa (Ac), and retroauricular crease (Ra), representing dry, moist, and sebaceous sites, respectively. The swab collections were conducted twice at a 2- to 3-month interval. DNA extracted from these swabs were used as a template for library construction; amplicon sequencing was performed using an Illumina MiSeq sequencer. The spacer reads were then trimmed to remove primer sequences; sequencing statistics before and after primer trimming are summarized in Data Set S1, sheet 5. The average lengths of the spacer reads after primer trimming were 29.9, 29.7, and 35.3 bp for S. equinus, S. thermophilus, and M. luteus, respectively, which were comparable to the typical spacer length and the spacer length observed in CRISPRCasFinder analysis (Data Set S1, sheet 3). These trimmed reads were then used as input for downstream analysis. After quality control implemented in QIIME 2 (45), we detected a total of 476,185 spacers and 664 unique spacers for S. equinus, 460,309 spacers and 759 unique spacers for S. thermophilus, and 286,968 spacers and 204 unique spacers for M. luteus from 25 samples.

**FIG 2:**
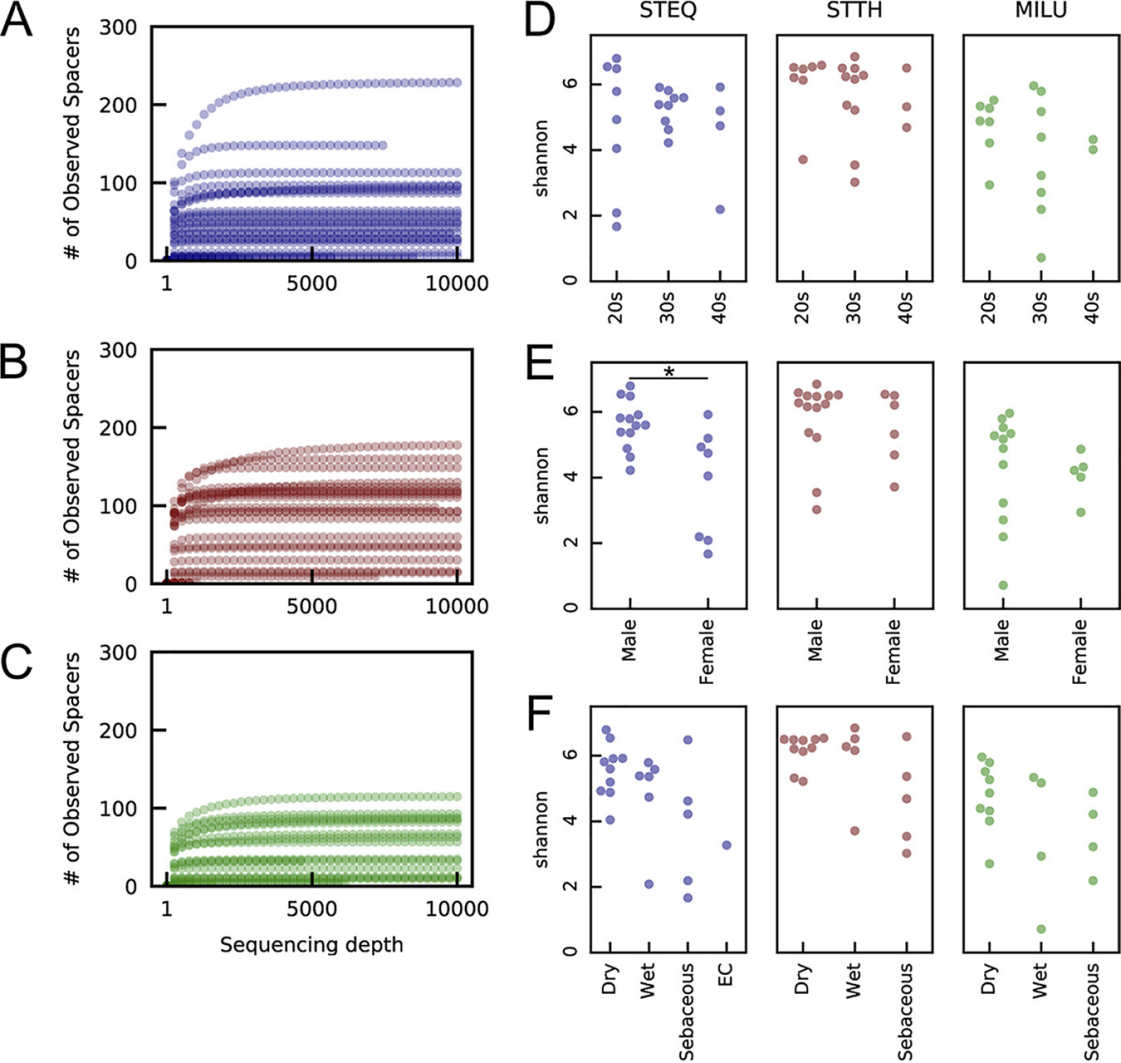
Alpha diversity of CRISPR spacer sequences in the skin microbiome. (A to C) Rarefaction curves based on the ASVs of spacer sequences were calculated using the q2-diversity plug-in in QIIME 2 with 100 iterations and a maximum sequence depth of 10,000. S. equinus CRISPR (A), S. thermophilus CRISPR (B), and M. luteus CRISPR (C) spacers. (D to F) The Shannon diversity index of each sample was calculated using the q2-diversity plug-in in QIIME 2. The Shannon indices of each sample group were compared using the Kruskal-Wallis test (*, *P* < 0.05). Comparisons were in terms of age range (D), gender (E), and site characteristics (F).

A rarefaction curve based on the observed amplicon sequence variants (ASVs) reached a saturation plateau at a sequencing depth of 4,000, indicating that the spacer diversities of all samples were well represented at a relatively low sequencing depth (Fig. 2A to C). Saturation of the rarefaction curve suggested low PCR error in the MiSeq results; therefore, an ASV table was used to evaluate spacer diversity rather than operational taxonomic unit (OTU) clustering (46). Samples were rarefied to 4,829 reads for normalization, and those with lower numbers of reads were discarded from downstream analyses. Richness and evenness of the spacer diversity in each sample were evaluated by calculating the Shannon diversity index. The medians of Shannon diversity indices, after rarefaction and removal of low-depth samples, were 5.27 (interquartile range [IQR], 4.32 to 5.81), 6.22 (IQR, 5.29 to 6.50), and 4.39 (IQR, 3.23 to 5.27) for S. equinus, S. thermophilus, and M. luteus, respectively. We also used Hill numbers, which reflect richness and evenness if plotted as a function of the parameter *q*, to estimate the diversity profile of each CRISPR array (47). The Hill number plot indicated that S. thermophilus had the highest spacer richness and evenness (see Fig. S3). There were no significant differences in Shannon indices of the S. thermophilus and M. luteus spacers when we grouped samples by gender, age, or site characteristics. In contrast, significant differences in Shannon indices of the S. equinus spacers were observed when samples were grouped by gender (Fig. 2D to F). These results indicate that the evenness and richness of spacer sequences in the skin microbiome are not affected by the environment from which the bacteria were collected.

10.1128/mSystems.01255-20.3 FIG S3 Hill numbers plotted as a function of the parameter. The Hill numbers were calculated using the library “vegan” in R. Download FIG S3, PDF file, 0.1 MB. Copyright © 2021 Toyomane et al. 2021 Toyomane et al. This content is distributed under the terms of the Creative Commons Attribution 4.0 International license.

**FIG 3:**
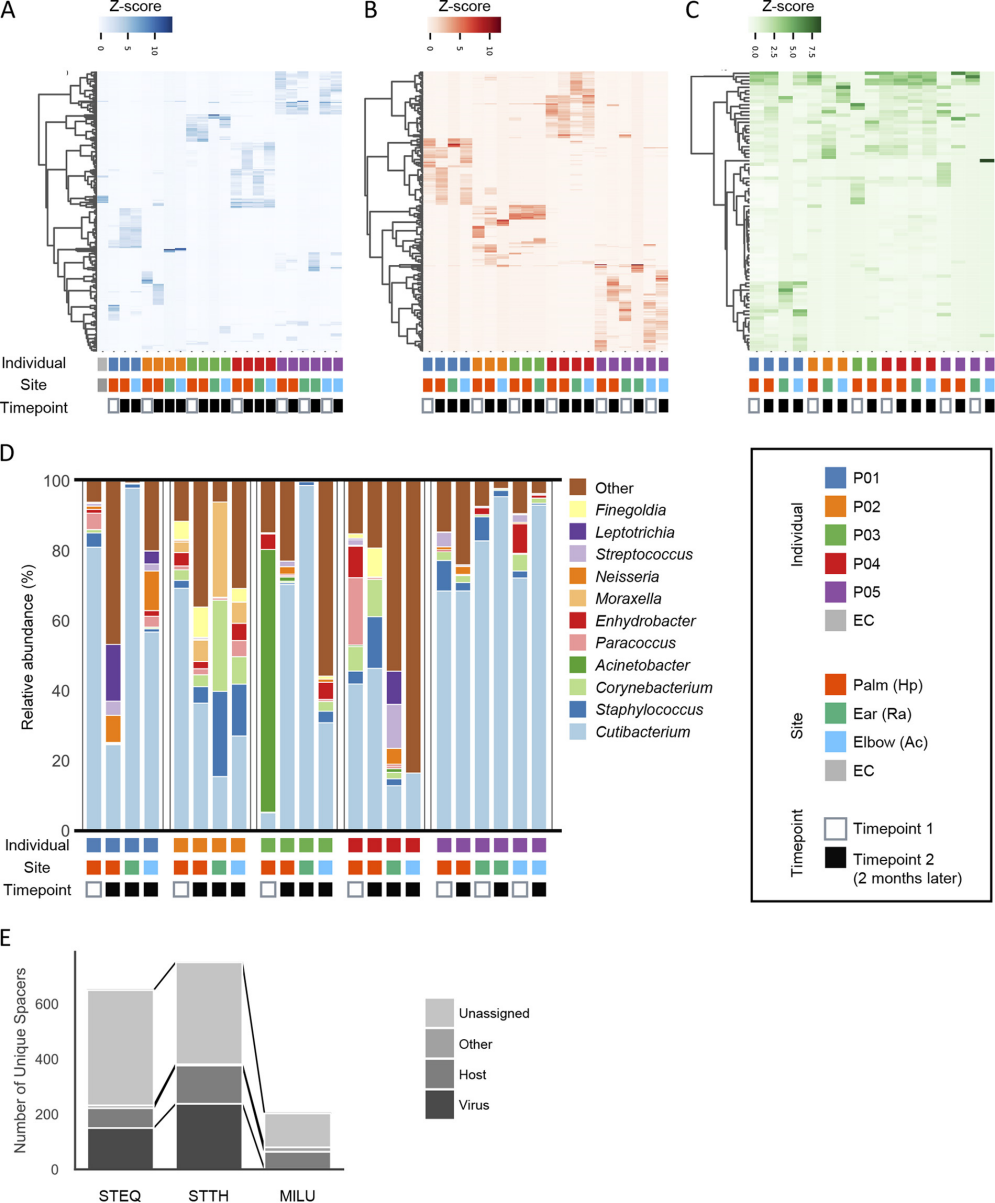
The pattern of CRISPR spacer and that of microbiome measured by 16S RNA in the skin microbiome. (A to C) Heat maps of the CRISPR spacers in the skin microbiome. Each row represents a unique spacer sequence ASV, and the columns represent samples from five individuals. Each row was clustered by the Wards method implemented in the Seaborn package. Each column was standardized by the Z-score. The ASVs that were observed equal or more than 100 times in total were used to generate the heat maps. S. equinus (A), S. thermophilus (B), and M. luteus (C) spacers. (D) Skin microbiome profile measured by 16S rRNA V1-3 sequencing. The compositions of the top 11 genera are shown. (A to D) Boxes below the graphs indicate sampled individual, site, and time point. (E) Numbers of unique spacer sequences and classification of the sequences were compared by CRISPRs. See also Data Set S1, sheets 6 to 8, for the detailed BLAST search results.

We then characterized the distribution of each spacer sequencers among the individuals to determine whether each spacer was specific to each individual or sample type. In accordance with a previous report (36), we found that the S. equinus or S. thermophilus spacer sequences were shared within individuals even for samples collected from different skin sites or at sampling time point (Fig. 3A and B), suggesting that these two CRISPRs are suitable for personal identification purposes. In contrast, the most abundant M. luteus spacers were shared between individuals, regardless of sample type (Fig. 3C). Although microbial communities harboring the CRISPR arrays sequenced in this study were obtained from the same environment, a high degree of interindividual CRISPR spacer dissimilarities were observed in S. equinus and S. thermophilus but not in M. luteus. These results suggest that the frequency of formational change in CRISPR arrays, including spacer acquisition or loss, differs among CRISPRs even in the same environment.

### CRISPR reconstruction from spacer amplicons.

While we targeted short reads that contain a single spacer in a read, we also obtained longer reads that contain two spacers in a read. Using these reads, we assembled the CRISPR locus from amplicons, as was done by Robles-Sikisaka et al. (38). Among the 136 assembled arrays, 35 arrays were found to share at least one spacer with another array; groups of arrays sharing spacers are shown in Fig. S4. Among them, the S. equinus CRISPR group 1 contains two identical CRISPRs from the same individual at different time points, suggesting a conserved CRISPR array on the skin. Furthermore, we observed that the CRISPR arrays shared identical spacers from the same individual at different sites on the skin at the same time point (e.g., S. equinus group 2, S. thermophilus group 6, and M. luteus group 1), from the same individual at the same site on the skin at different time points (e.g., S. equinus group 3, S. thermophilus group 4, and M. luteus group 2), or even from different individuals (e.g., S. equinus group 1, S. thermophilus group 1, and M. luteus group 1), suggesting both conservation and diversity of these CRISPRs. However, we could not determine whether these partial matches are derived from the acquisition/loss of spacers or assembling errors, especially when the spacers of the array ends did not match. Nevertheless, we observed the internal loss of spacers in some groups (S. equinus groups 1 and 6 and S. thermophilus group 7), suggesting that amplicon sequencing can be used to trace the phylogenetical relationship of CRISPR arrays in the environment.

10.1128/mSystems.01255-20.4 FIG S4 Comparison of putative CRISPR arrays reconstructed from amplicon sequencing. The S. equinus (A), S. thermophilus (B), and M. luteus (C) CRISPR arrays were reconstructed from spacer amplicons and analyzed by CRISPR Visualizer (71). CRISPR arrays sharing spacers were grouped together. Each colored box represents a spacer. Gray boxes with “x” represent missing spacers. Note that similar colors do not indicate similar sequences. Download FIG S4, PDF file, 0.1 MB. Copyright © 2021 Toyomane et al. 2021 Toyomane et al. This content is distributed under the terms of the Creative Commons Attribution 4.0 International license.

### Assessment of skin bacterial community by 16S rRNA sequencing.

We also assessed the structure of the skin microbiome using 16S rRNA V1-3 amplicon sequencing with a MiSeq sequencer. We obtained 1,530,894 ASVs from 25 samples after read filtering (mean, 61,235.76 reads/sample; IQR, 33,802 to 90,173 reads/sample). A total of 2,358 unique reads with a median length of 476 bp (IQR, 432 to 487 bp) after primer trimming were obtained. Rarefaction analysis indicated that the number of observed species did not plateau at 5,000 reads, suggesting that 16S rRNA analysis requires a greater sequencing depth than spacer sequencing (Fig. 2A to C; see also Fig. S5). This relatively high number of required reads for 16 rRNA sequencing is consistent with a previous report that analyzed the V4-5 region (48). As shown in Fig. 3D, the most common genus in the skin microbial community was *Cutibacterium*, which is represented by C. acnes, the most abundant species on human skin (2, 3). Although *Moraxella* was specifically and stably observed in individual P02, other abundant genera were observed in all individuals (e.g., *Cutibacterium*, *Staphylococcus*, and *Corynebacterium*) or specifically but not stably observed in an individual (e.g., Acinetobacter in individual P03). Thus, compared with CRISPR spacer diversity, the composition of abundant genera in the skin microbiome is likely insufficient for personal discrimination.

10.1128/mSystems.01255-20.5 FIG S5 Rarefaction curve analysis of 16S rRNA V1-3 sequencing data. Rarefaction curves based on the ASVs of spacer sequences were calculated using the q2-diversity plug-in in QIIME 2 with 100 iterations and a maximum sequence depth of 40,000. Download FIG S5, PDF file, 0.1 MB. Copyright © 2021 Toyomane et al. 2021 Toyomane et al. This content is distributed under the terms of the Creative Commons Attribution 4.0 International license.

### Putative protospacers of the spacers reads.

Spacers of a CRISPR loci are acquired from previously encountered foreign genetic elements by host bacteria (16). To identify possible protospacers, which are the origins of spacers, spacer reads were subjected to BLAST searches (Data Set S1, sheets 6 to 8; Fig. 3E). For streptococcal CRISPRs (S. equinus and S. thermophilus), 27.7% of spacer sequences were previously found in viruses, the sources of new spacers. In contrast, only one virus sequence was identified from M. luteus spacers. Some sequences were those of host bacteria: 15.1% for streptococcal spacers and 31.0% for M. luteus. These host-derived spacers may target plasmids or CRISPR loci or are self-targeting (49). Interestingly, 34 sequences (16.7% of total unique spacers) of M. luteus spacers derived from the “host” were sequences of single strains of M. luteus (GenBank CP025616.2). Spacers of the strain were regularly ordered with consistent repeats in the same direction, indicating that they were located on a single CRISPR locus. In total, 56.8% of the spacers were previously unknown sequences. This high percentage of unassigned reads was expected, as most phages have no homologous sequence in public databases (50). Altogether, the sequences identified by our spacer sequencing are likely derived from actual environmental CRISPRs, supporting the validity of our proposed method.

### Individual-specific spacer patterns.

**FIG 4:**
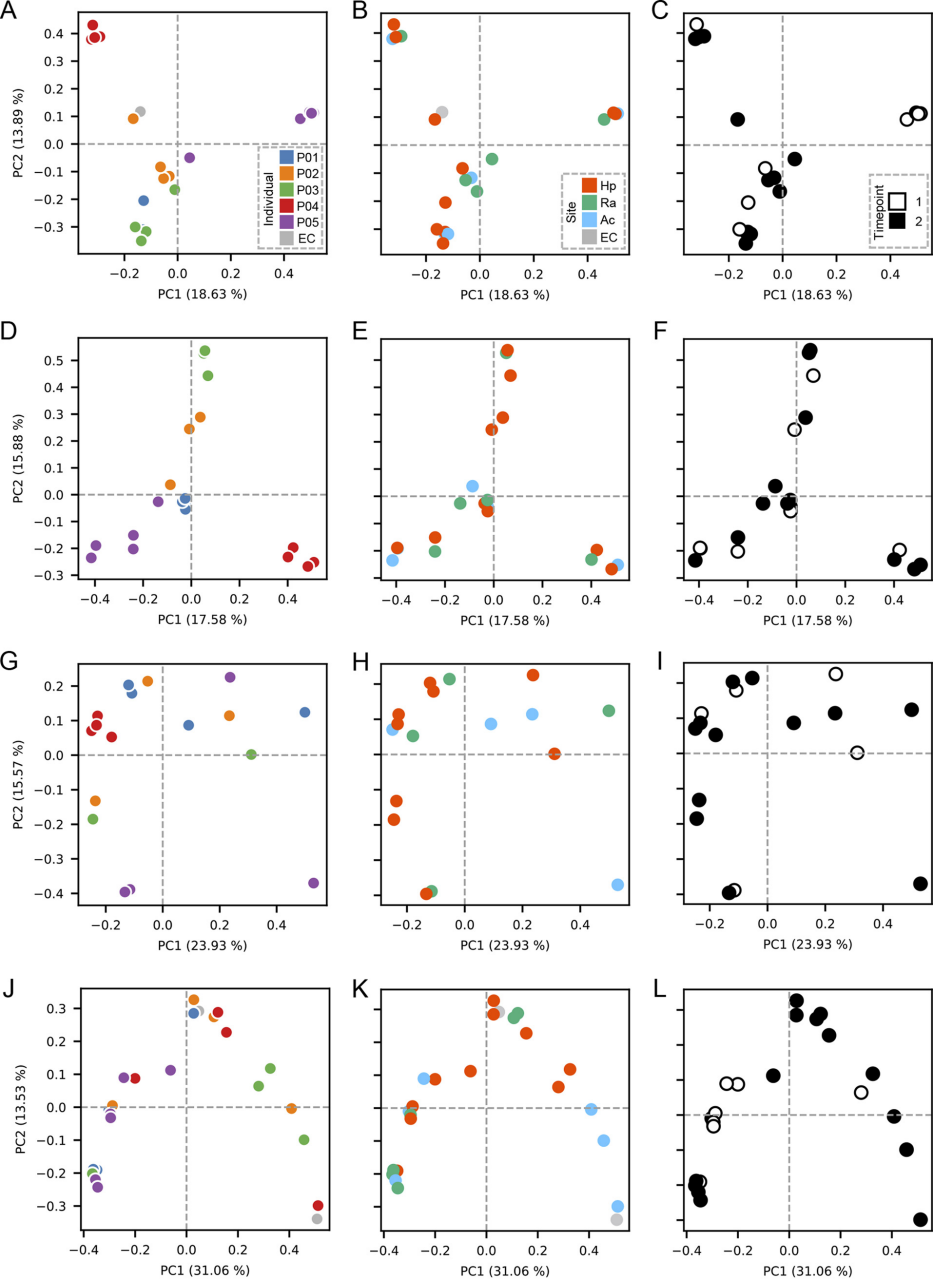
Principal-coordinate analysis of the spacers and 16S rRNA. PCoA plots of the S. equinus (A to C), S. thermophilus (D to F), and M. luteus (G to I) spacer sequences as well as the 16S rRNA V1-3 sequences (J to L). The Bray-Curtis dissimilarity index was used as a metric for PCoA. Each data point is colored according to the individual (A, D, G, and J), skin site (B, E, H, and K), or time point (C, F, I, and L).

**FIG 5:**
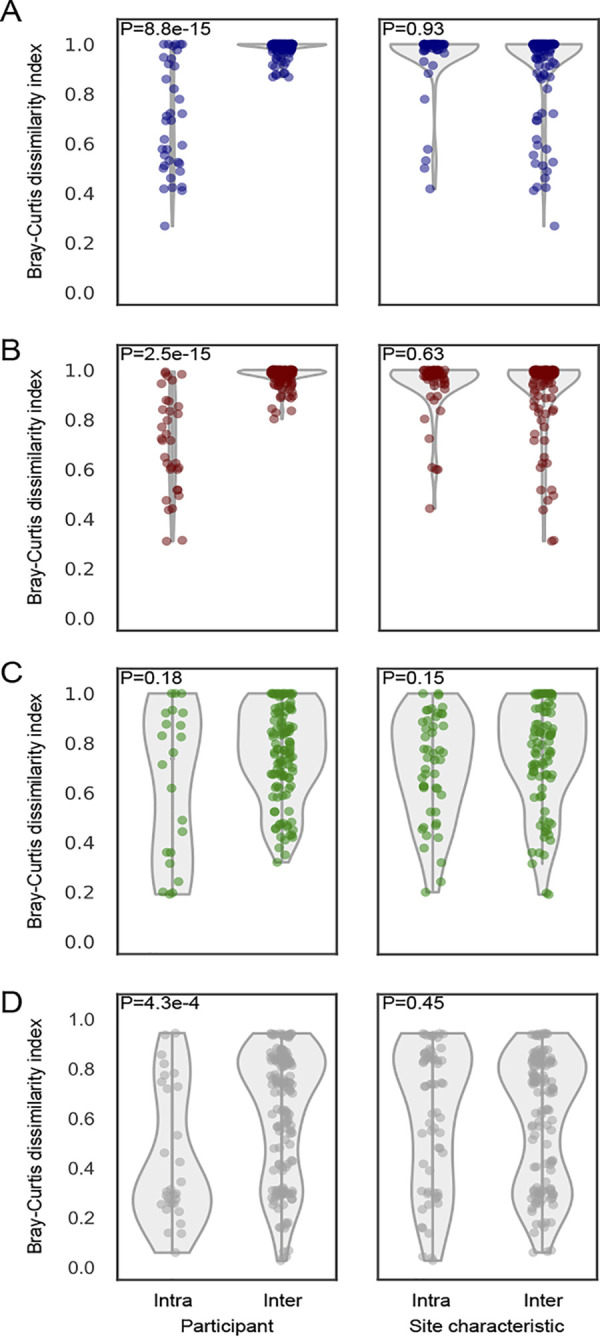
CRISPR spacers are individual specific rather than site-specific. (A to D) Violin plots of the Bray-Curtis dissimilarity indices within (intra) or between (inter) individuals/host site of S. equinus (A), S. thermophilus (B), M. luteus (C), and 16S rRNA V1-3 (D). The Bray-Curtis dissimilarity indices were compared using the Mann-Whitney U test.

The Bray-Curtis dissimilarity index (BC) for CRISPR spacers, which represents ecological similarities of the spacer community between samples, was calculated and compared with that of 16S rRNA V1-3 sequences; BC equals 1 if the two communities do not share any spacers, while BC equals 0 if the two communities are identical. Principal-coordinate analysis (PCoA) of BC of S. equinus and S. thermophilus spacers showed strong clustering of the samples from the same individuals, indicating that the diversity of these spacers was more reflective of the host than of sample type (Fig. 4A to F). In contrast, the PCoA plots of BC for neither M. luteus spacers nor 16S rRNA showed any specific clustering (Fig. 4G to L). Notably, S. equinus and S. thermophilus spacers were significantly more individual specific than site specific (Fig. 5A and B). No significant differences were observed in M. luteus spacers when the dissimilarities were compared among or between individuals (Fig. 5C), indicating the differences in interindividual dissimilarities of spacers among these CRISPRs. For S. equinus and S. thermophilus, none of the BCs calculated from a pair of interindividual samples was <0.8, indicating that these high interindividual dissimilarities may sufficiently discriminate two individuals. This high dissimilarity in interindividual samples was not observed for 16S rRNA sequencing (Fig. 5D), suggesting that interindividual dissimilarity of microbiomes measured by 16S rRNA sequencing does not have sufficient discriminating power for forensic cases. Collectively, these results indicate that S. equinus and S. thermophilus spacer diversity in the skin microbiome is highly personalized compared to the microbiome diversity measured by 16S rRNA V1-3 sequencing; thus, this individuality can be used as a discriminating marker.

### Personal identification by a set of predictive spacers.

**FIG 6:**
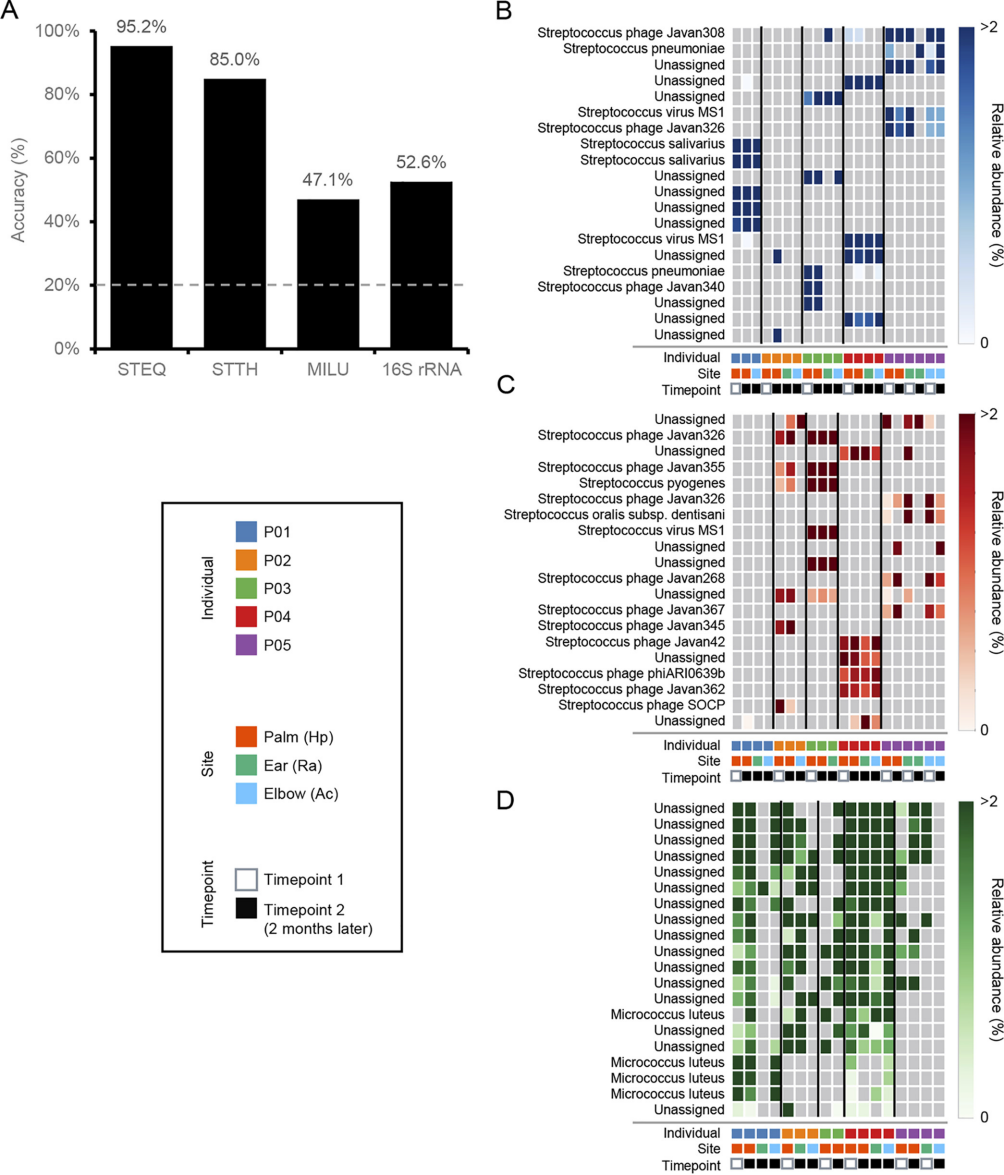
Identification of the predictive spacer sequences for personal identification. (A) Comparison of accuracy results using S. equinus, S. thermophilus, and M. luteus CRISPRs and 16S rRNA data. The accuracy results were obtained using 20 features selected by SVM-RFE. After feature selection, SVM with leave-one-out cross-validation was performed to classify the samples from the individuals. Baseline accuracy is indicated by gray dashed line. Heat maps of the relative abundance of the 20 selected features for S. equinus (B), S. thermophilus (C), and M. luteus (D). Data points with absent features are grayed out. The annotation of each spacer sequence was based on the BLAST search described in Data Set S1, sheets 6 to 8.

To test whether a set of discriminant spacers can aid identification of the personal microbiome, we utilized support vector machine based on recursive feature elimination (SVM-RFE; see Materials and Methods for details), a machine learning technique used in bioinformatics research to predict sample type with a small number of features (51, 52). Using SVM-RFE, we successfully selected 20 predictive features for each CRISPR and 16S rRNA (Fig. 6). For S. equinus CRISPR, 20 spacers achieved 95.2% accuracy for personal identification, which was the highest performance in our data sets. Although 20 spacers from S. thermophilus achieved 85.0% accuracy, the accuracy rates obtained using selected features of M. luteus or 16S rRNA were 47.1% and 52.6%, respectively. The relative abundance heat maps of the 20 spacers showed that the presence of spacers in streptococcal CRISPRs had a distinct and specific pattern for each individual (Fig. 6B and C), whereas M. luteus spacers did not (Fig. 6D). These individual specific spacers likely contributed to the higher accuracy. Therefore, the spacer pattern of even a single CRISPR array, such as S. equinus CRISPR, may sufficiently discriminate personal microbiome signatures for future personal identification.

## DISCUSSION

CRISPR typing has been used to discriminate bacterial species at the strain level (21–25, 27). Here, we proposed metagenomic CRISPR typing as a new personal microbiome typing method, instead of conventional 16S rRNA V1-2/V4 sequencing (1, 6, 53). Regardless of previous studies that utilized amplicon sequencing for evaluation of spacer diversity in oral, skin, or lake environments (20, 32, 36–38), the high interindividual diversity of spacer sequences on human skin is still largely unknown, given that the specific CRISPR arrays in skin environments commonly shared among individuals have not been clarified. In this study, we identified putative CRISPRs shared between the skin microbiomes of different individuals by reconstructing CRISPR arrays using publicly available metagenome data sets (Fig. 1). We further examined the distribution of spacers in the skin microbiome by amplicon sequencing and found that CRISPR diversity was highly person specific regardless of skin site, unlike the skin microbiome structure assessed by 16S rRNA V1-3 sequencing (Fig. 3 to 5).

We initially identified the consensus repeats of CRISPR arrays conserved among individuals by metagenomic reconstruction of CRISPR arrays. The repeats of CRISPRs derived from S. equinus, N. polysaccharea, S. thermophilus, Veillonella parvula, Aggregatibacter aphrophilus, and S. wolfei were observed in 14 of 15 individuals (Fig. 1B), suggesting that CRISPR arrays with those repeats are widely spread among the human population. This observation is partially in agreement with a study by Robles-Sikisaka et al. (38), in which spacer diversity of *Streptococcus*-derived CRISPRs in the human skin microbiome was investigated by amplifying the spacer regions with primers targeting the repeat derived from S. equinus.

Interestingly, CRISPR arrays derived from *C. acnes*, which is the most abundant microorganism in the skin microbiota (2, 3), were rarely detected in the present study. This discrepancy may be attributed to the scarcity of CRISPR arrays in populations of *C. acnes*, as only one lineage of *C. acnes* among the three major groups harbors CRISPR arrays, and those in the organism harbor a limited number of spacers (1 to 9 spacers, with a mean of 3.31) (54–56). Fitz-Gibbon et al. reported that only two of the top 10 most abundant *C. acnes* ribotypes are associated with CRISPR-Cas and that the CRISPR-encoding ribotypes are not major in >80% of individuals (55), supporting the limited observation of the *C. acne* CRISPRs. In contrast, the repeats detected in our metagenomic CRISPR reconstruction were derived from minor genera in the skin metagenomic data, such as *Chryseobacterium* and *Aggregatibacter*, which comprised <0.10222% of reads in the metagenomic data set produced by Oh et al. (2). The present study is the first to evaluate the ecology of CRISPRs in the skin microbiome, identifying previously unknown CRISPRs and evaluating CRISPR abundance in the human-associated microbiome, providing insight into the nature of the skin microbiome.

Spacer sequences in the human skin microbiome were then amplicon sequenced to identify the CRISPRs that are shared by multiple individuals and those that are highly individual specific. Although the number of participants was limited, we successfully amplified the spacers from all five individuals, supporting the notion that putative CRISPRs in the metagenomic data sets were conserved among individuals. As hypothesized, the diversities of the S. equinus and S. thermophilus spacers were highly distinct, whereas major spacers of the M. luteus CRISPR were shared between individuals (Fig. 3 and 6), indicating that S. equinus and S. thermophilus are good candidates for future personal identification markers. Unexpectedly, even major spacers were shared between samples collected at different time points from the same individual, suggesting that spacer diversity was stable over 2 months (Fig. 3 and 6). Yang et al. demonstrated that 16S rRNA V1-2 sequencing is not sufficient to discriminate personal microbiomes (15). Our results also support that skin microbiome composition measured by 16S RNA sequencing is insufficient for personal discrimination (Fig. 5 and 6). In contrast to bacterial communities, membership of bacteriophage communities was mostly individual specific (20). The membership of CRISPR spacers also reflects this individual specificity of bacteriophages, which likely contributes to high interindividual dissimilarity. This speculation was supported by the observation of phage-derived sequences in the predictive spacers of streptococcal CRISPRs (Fig. 6B and C). Thus, polymorphisms of CRISPR spacers, rather than conventional 16S rRNA, may be useful for discriminating between suspects in criminal investigations.

Two CRISPR arrays identified, S. equinus and S. thermophilus, were derived from *Streptococcus* species, which are also known as the S. thermophilus CRISPR-Cas9 system in CRISPR1 and CRISPR3 loci, respectively. The CRISPR loci of S. thermophilus have been intensively studied in both evolutionary and genome editing perspectives (43). The CRISPR arrays of S. thermophilus are classified into four CRISPRs: CRISPR1 (S. equinus), CRISPR2, CRISPR3 (S. thermophilus), and CRISPR4. Paez-Espino et al. revealed that spacer acquisition ability differs between them, as CRISPR1 integrated new spacers at a high frequencies, while CRISPR2 and CRISPR4 acquired no spacers in 232 days (57). In contrast to *Streptococcus* CRISPRs, which integrate a new spacer frequently, the spacer integration of M. luteus CRISPR has not been investigated, but the *cas* genes of M. luteus strain SGAir0127 were classified into the type I CRISPR-Cas system by CRISPRCasFinder. Although M. luteus is also a major member of the skin microbiome (2), the only available M. luteus CRISPR array in the NCBI database was that of strain SGAir0127, a strain isolated from indoor air samples (58). Though the nature of M. luteus CRISPR is unknown, we speculated that it lost its spacer acquisition ability during its evolution.

In this study, we focused on the diversity of spacers on a community level and did not comprehensively evaluate the temporal changes of single CRISPR arrays. The spacer acquisition ability of the streptococcal CRISPR-Cas system, which may contribute to the high interindividual spacer dissimilarity observed here, should be further assessed using other technologies, such as CRISPR locus PCR with the primer pair flanking an entire CRISPR region (26, 28–30). Nonetheless, we successfully reconstructed partial or entire CRISPR arrays from amplicon reads (see Fig. S4 in the supplemental material), suggesting that amplicon sequencing may be utilized to phylogenetically analyze CRISPR loci of interest, while technical optimizations, such as utilizing size selection during library construction and/or other sequence chemistries such as the paired-end sequencing of 600 cycles, will improve the shortcomings of our methodology on the CRISPR locus reconstruction analysis.

Although the resolution of CRISPR typing is sufficiently high for strain discrimination, other typing methods such as multilocus sequence typing, variable number tandem repeats typing (VNTR-typing) or 16S ribotyping can also be used to discriminate specific bacterial strains (55, 59–61). Recent studies also utilized such high-resolution typing methods in metagenomic contexts to characterize the compositions of specific species or bacterial communities (15, 62, 63). High interpersonal diversity of spacer sequences measured in this study demonstrate its potential for application in future forensic cases; however, it should be compared with other typing methods, such as *C. acnes* ribotyping. Importantly, the diversities of CRISPR arrays were similar between different skin sites from the same individual (Fig. 3, 4, and 6), suggesting that CRISPR spacers are shared among the skin sites. This spatial stability of CRISPR typing may be an advantage against *C. acnes* ribotyping, in which only palm samples were analyzed (15).

Though our results suggest that spacer diversity is highly personalized and useful as a new marker for personal identification, some aspects regarding the real-life applicability of this approach require further analysis. For example, we did not use our approach to test the microbiome on the surface of an object, such as a phone, a keyboard, or shoes, touched by a person of interest (suspect/victim) (1, 6, 15, 64). Another limitation of our study is the small sample size. Although we demonstrated that spacer diversity was sufficiently individual specific, the application in forensic cases requires population-wide validation to assess their validity and reliability. In addition, the performance in terms of personal identification was evaluated only for comparing the CRISPR-based and 16S rRNA-based approaches. Therefore, the score obtained in our study does not predict the performance of CRISPR typing on personal identification in a population, although we obtained 95.2% accuracy using S. equinus as a marker. By confirming the accuracy of CRISPR-based personal identification using mock forensic samples and conducting population-wide surveys in future studies, CRISPR-based microbiome analysis can be applied in actual forensic cases.

Although further developmental validation is required, the highly personalized diversity of CRISPR spacers in the human skin microbiome is an attractive tool for personal identification. Though the viral community is more personalized than the bacterial community (19, 20, 38), the lack of a common marker for characterizing the virome complicates its use for personal identification. In this study, the spacer sequences of CRISPRs were utilized as a proxy for the virome. This approach may also be utilized to better understand the ecology of the skin microbiome and explore the complex relationship between the human skin microbiome and diseases such as atopic dermatitis (65). The present study suggests that the diversity of the CRISPR spacer sequences in the skin microbiome can be used as a new marker for personal identification.

## MATERIALS AND METHODS

### Sample collection.

Five individuals were asked to participate in this study, and swab samples were collected for spacer sequencing. The participants provided information regarding their gender, age range, systemic antibiotics usage in the past 6 months, steroid usage in the past 6 months, skincare product usage in the past 24 h, and handwashing in the past 2 h. Data Set S1, sheet 9, in the supplemental material summarizes the detailed information of samples. Skin surfaces (approximately 5 by 5 cm) were swabbed with cotton-tipped swabs soaked in water. The Hp, Ac, and Ra, representing dry, moist, and sebaceous sites, respectively, were selected for swabbing (2). For the initial sampling (time point 1), only an Hp sample was obtained, except for one individual (P05). Two to 3 months later (time point 2), three skin sites (Hp, Ac, and Ra) were sampled for all individuals. After swabbing, the cotton tips were separated and placed in 2-ml tubes. A premoistened swab tip placed directly in the 2-ml tube was used for a blank for DNA extraction (EC). Samples were stored at −20°C before DNA extraction. All procedures involving human participants were approved by the Institutional Ethics Committee of the National Research Institute of Police Science.

### Metagenomic data sets.

Metagenomic data sets from the skin of the palm were downloaded from the European Nucleotide Archive in fastq format. The samples used in this study have been registered under BioProject PRJNA46333. Data collection methods were performed in accordance with the method previously reported by Oh et al. (2). Samples containing multiple runs were merged before assembly.

### Identification of CRISPR arrays using metagenomic data sets and isolation of CRISPRs conserved across multiple individuals.

The scheme for CRISPR reconstruction from the metagenomic reads and isolation has been summarized in Fig. S6. Reads were filtered using Trimmomatic 0.39 to remove low-quality reads (66), bases with low-quality scores (Q < 20) were trimmed from both ends, and short reads (<50 bp) were removed. As the file was too large to assemble, 8 × 10^7^ reads (22%) were subsampled from the largest data set (SRX743731) using seqtk (https://github.com/lh3/seqtk). *De novo* assembly of the metagenome was performed using MEGAHIT v1.1.4 (39). The data sets were assembled with default parameters, except that the k-mer sizes were fixed as –k-list 29,39,59,79,99. Output contigs were analyzed using the PILER-CR v1.06 with default parameters to detect the CRISPR arrays (40). Each repeat in a CRISPR array detected by the PILER-CR was converted to a single read. All reads were then duplicated, and one of the read pairs was reverse complemented, as the orientation of repeat sequence was unclear.

10.1128/mSystems.01255-20.6 FIG S6 Schematic of CRISPR reconstruction from the metagenomic reads and identification of shared CRISPR repeats. *De novo* assembly of the metagenome was performed with MEGAHIT v1.1.4. Output contigs were analyzed using PILER-CR v1.06 with default parameters to detect the CRISPR arrays. All reads were then duplicated, and one of the read pairs was reverse complemented. Repeat reads were then dereplicated and clustered into OTUs by QIIME 2 v2019.7. The duplicated reverse complement repeat reads were discarded after OTU clustering. Download FIG S6, PDF file, 0.2 MB. Copyright © 2021 Toyomane et al. 2021 Toyomane et al. This content is distributed under the terms of the Creative Commons Attribution 4.0 International license.

Repeat reads were then analyzed using QIIME 2 v2019.7 (45). First, dereplication and *de novo* OTU clustering at 90% were performed using the q2-vsearch plug-in (67). The repeat reads were detected in fewer than two individuals, and the duplicated reverse complement reads were discarded. The representative CRISPR repeats of each OTU were annotated by BLAST using default settings (68). The subject with the highest max score was defined as the host species. Since CRISPRs are mobile genetic elements, multiple species were often found to share the same repeat (69); in such cases, one representative species was recorded. Eventually, heat maps of the repeat reads on the OTU table were constructed using the heatmap visualizer of the q2-feature-table plug-in (70). The type and orientation of CRISPRs were determined by a search for the same repeats in CRISPRCasdb (44).

We also utilized CRISPRCasFinder to identify *cas* genes associated with the CRISPR locus found in metagenomic contents (42). The contigs assembled by MEGAHIT were used as initial inputs. We initially extracted the contigs that contained CRISPRs with evidence level 4 and were >1,000 bp in length using CRISPRCasFinder with default settings. The evidence level was determined by CRISPRCasFinder. Subsequently, the extracted contigs were subjected to CRISPRCasFinder again to determine if the contigs contained *cas* genes and to subtype the identified *cas* genes based on the nomenclature proposed by Makarova et al. (41) using CRISPRCasFinder with the -cas parameter. Three representative contigs containing S. equinus CRISPR arrays were compared with the genomes of *Streptococcus* strains (Data Set S1, sheet 10) using CRISPR Visualizer (71).

### Measurement of DRs in the metagenomic data set by BLAST search.

The number of reads containing each repeat in a data set was measured using BLAST. The sequences of repeats were used as queries, and the skin metagenome data sets in the Sequence Read Archive (SRA) of NCBI were used as subjects. The data sets used as subjects were identical to those used in the CRISPR reconstruction, except for samples from nares or cheek. Two samples (SRX740760 and SRX740761) from nares or cheek, representing a moist or oily site, respectively, of the same individual (HV07) were also included to evaluate repeat variation at different skin sites from an individual. Default parameters were used for the short-read search except that the maximum number of target sequences was set as 5,000. Reads with >90% identity and >90% query cover were included in the downstream analysis. A hierarchically clustered gradient heat map of repeat frequency was plotted using the clustermap method in the Seaborn package 0.9.0 (https://seaborn.pydata.org/generated/seaborn.clustermap.html) in Python 3.6.8.

### DNA extraction.

Genomic DNA was extracted from swabs using the DNeasy PowerSoil kit (Qiagen, Aarhus, Denmark) with some minor modifications (1, 72). Briefly, frozen cotton tips were transferred to the bead tube provided with the DNeasy PowerSoil kit, followed by incubation at 70°C for 15 min after the addition of the C1 solution. Tubes were then horizontally shaken at 3,200 rpm for 2 min on a bead beater-type homogenizer μT-12 (TAITEC, Saitama, Japan). Remaining steps were performed according to the manufacturer’s instructions. Genomic DNA was eluted in 100 μl of the C6 solution.

### Amplification and sequencing of spacers.

To amplify the spacer regions of the putative CRISPRs, three pairs of primers were designed based on the specificity of the CRISPR repeat, namely, S. equinus, S. thermophilus, and M. luteus (Data Set S1, sheet 11). These primer pairs are specific to the 3′ region of each CRISPR repeat so that the primer pair flanks a spacer. The length of primers was determined to meet the melting temperature requirement on the Illumina 16S Metagenomic Sequencing Library Preparation protocol (https://www.illumina.com/content/dam/illumina-support/documents/documentation/chemistry_documentation/16s/16s-metagenomic-library-prep-guide-15044223-b.pdf). PCR was performed in a 50-μl reaction volume using the KOD FX (Toyobo, Osaka, Japan). Each reaction mixture consisted of 1× PCR buffer for KOD, 0.4 mM deoxynucleoside triphosphate (dNTPs), 0.3 μM each primer, 1.0 U of KOD FX, and 2 μl of the DNA template. EC and a nontemplate control (NTC) for PCR were included as negative controls. The following conditions were used for amplification: 2 min at 94°C, 35 cycles of denaturation at 95°C for 30 s, annealing at 60°C for 30 s, and extension at 72°C for 30 s, followed by final extension at 72°C for 5 min. Next, 25 μl of the products was purified using 1.8× volume of the Agencourt AMPure XP kit (Beckman Coulter, Brea, CA) twice and washed using freshly prepared 80% ethanol. DNA was eluted in 52.5 μl of 10 mM Tris (pH 8.5) and 0.1% Tween 20 elution buffer. If low-molecular-weight bands were observed using agarose gel electrophoresis, additional purification was performed using 1.8× volume of the Agencourt AMPure XP kit. Purified DNA was indexed using the Nextera XT DNA index kit (Illumina, San Diego, CA) and KOD FX in a reaction volume of 50 μl. Each reaction mixture consisted of 1× PCR Buffer for KOD, 0.4 mM dNTPs, 4.5 μl of each index primer, 1.0 U of KOD FX, and 5 μl of the purified DNA as the template. The index PCR product was purified using 1.8× volume of the Agencourt AMPure XP kit and eluted in 27.5 μl of 10 mM Tris (pH 8.5) and 0.1% Tween 20 elution buffer. The indexed libraries were quantified using GenNext NGS library quantification kit (Toyobo) and then diluted to 4 nM with an elution buffer. If the library concentration was less than 4 nM, the library was pooled with other libraries without dilution. Denaturing and final dilution were performed according to the manufacturer’s instructions. Each run included >15% of the PhiX control v3 (Illumina) to improve sequencing quality. Paired-end sequencing of 300 cycles (2 × 151) was performed on a MiSeq platform (Illumina) with the MiSeq v2 chemistry, according to the manufacturer’s instructions.

### Amplification and sequencing of 16S rRNA.

Sequence libraries of the 16S rRNA were prepared according to the Illumina 16S metagenomic sequencing library preparation protocol with some modifications. The primer pair targeting the V1-V3 region (73, 74) was used in this study (Data Set S1, sheet 11). PCR was performed in a 25-μl reaction volume using the KAPA HiFi HotStart ReadyMix kit (KAPA Biosystems, MA). Each reaction consisted of 1× KAPA HiFi HotStart ReadyMix, 0.3 μM each primer, and 2 μl of the DNA template. The following conditions were used for amplification: 5 min at 95°C, 25 cycles of denaturation at 95°C for 30 s, annealing at 62.3°C for 30 s, and extension at 72°C for 30 s, followed by final extension at 72°C for 5 min. Then, 25 μl of the products was purified using 0.8× volume of the Agencourt AMPure XP kit and washed using freshly prepared 80% ethanol. DNA was eluted in 52.5 μl of 10 mM Tris-HCl (pH 8.5) and 0.1% Tween 20 elution buffer. Purified DNA was indexed using the Nextera XT DNA index kit (Illumina) and the KAPA HiFi HotStart ReadyMix kit in a reaction volume of 50 μl. Each reaction mixture consisted of 1× KAPA HiFi HotStart ReadyMix, 5 μl of each index primer, and 5 μl of the purified DNA as the template. The index PCR product was purified using 1.1× volume of the Agencourt AMPure XP kit and eluted in 27.5 μl of 10 mM Tris (pH 8.5) and 0.1% Tween 20 elution buffer. The indexed libraries were quantified by the GenNext NGS library quantification kit and diluted to 4 nM with elution buffer. If the library concentration was less than 4 nM, the library was pooled with other libraries without dilution. Denaturing and final dilution were performed according to the manufacturer’s instructions. Each run included >15% of the PhiX control v3 to improve sequencing quality. Paired-end sequencing of 600 cycles (2 × 301) was performed on a MiSeq platform (Illumina) with MiSeq v3 chemistry, according to the manufacturer’s instructions.

### Data processing for spacer analysis.

Initial quality controls were carried out using the CLC Genomics Workbench 12.0.2 (Qiagen). After low-quality sequences (Q < 20) and primer sequences were removed, reads shorter than 20 bp or longer than 40 bp were filtered. The remaining spacer sequences were then analyzed using QIIME 2 v2019.7 (45). Spacer reads were denoised and dereplicated with the denoise-paired method of the q2-dada2 plug-in (75) with min_fold_parent_over_abundance = 32; the resulting ASV tables were further analyzed. Rarefaction curves were created with the q2-diversity plug-in with 100 iterations and 40 steps at a maximum depth of 10,000 reads. Based on rarefaction curve analysis, each sample was rarefied to 4,829 reads for normalization in further analysis, and samples with fewer than 4,829 spacer ASVs were omitted from the calculation (76). The EC and NTC were removed at this step; however, EC was included in S. equinus spacer analysis. Shannon indices were calculated in bits with the q2-diversity plug-in, and Kruskal-Wallis tests were performed to compare Shannon indices between samples (77). For calculation of the beta-diversity index, the Bray-Curtis dissimilarity index was calculated by the q2-diversity plug-in and used for PCoA plots. Mann-Whitney U test was calculated using the “stats.mannwhitneyu” function of the python library “SciPy” (version 1.3.0) to compare the Bray-Curtis dissimilarity indices. Hill numbers were calculated using the library “vegan” (version 2.5-4) in R (version 3.4.4) (78).

To compare the CRISPR arrays in the samples, we reconstructed the arrays from spacer amplicons. The trimming and assembling described below were performed using the CLC Genomics Workbench 12.0.2. First, reads that were longer than 100 bp were extracted after low-quality sequences (Q < 20) were removed so that reads contained two spacers per read. Then, these reads were *de novo* assembled by “Map reads back to contigs” mode with a length fraction of 0.8 and similarity fraction of 0.9. These assembled contigs were then compared and visualized using CRISPR Visualizer (71).

The spacer sequences were subjected to BLAST analysis with default settings. If the reads had E values of less than 0.01, they were further classified. If the description included “phage” or “virus,” the query was classified as “virus.” If the description included name of host genus (“streptococcus” for S. equinus or S. thermophilus and “micrococcus” for M. luteus), the query was classified as “host.” Remaining queries with significant hits were classified as “other.”

To identify a subset of predictive spacers and to improve interpretability of the classification, we performed feature selection using support vector machine based on recursive feature elimination (SVM-RFE). SVM-RFE is a machine-learning technique used to select a subset of features (e.g., genes associated with a specific disease) which are relevant to sample types from a large set of features, by recursively ranking the features and eliminating irrelevant features (79). In this study, SVM-RFE was implemented using the python library “Scikit-learn” (version 0.22.1) (80). For SVMs, the linear kernel was used with default parameters. Using SVM-RFE, we selected 20 ASVs from the data set, including all samples from an individual. Then, using the selected 20 features, we predicted the individuals by linear-kernel SVM using leave-one-out cross-validation to measure predictive accuracy. However, we did not perform nested cross-validation to optimize the selection of ASVs used in the linear-kernel SVM. This is because it is difficult to split the data twice for inner and outer cross validation due to the small sample size, which may cause overfitting of the data.

### Data processing for 16S rRNA analysis.

Reads were analyzed using QIIME 2 v2019.7 (45). Initially, 16S rRNA reads were denoised and dereplicated with the denoise-paired method of the q2-dada2 plug-in (75). The parameters of trim_left_f equals 20 and trim_left_*r* equals 20 were set to trim primer sequences. The resulting ASV tables and representative reads were further analyzed. Representing reads were annotated by classify_sklearn methods of the q2-feature-classifier plug-in with the SILVA 16S rRNA database v132 (80, 81). We observed contaminants derived from *Sphingomonadaceae* and *Pseudomonadaceae*, which are known as common contaminants (82), in both swab samples and NTC due to low biomass. Therefore, we removed reads annotated as *Sphingomonadaceae* or *Pseudomonadaceae* from the tables and representative reads using filter-table and filter-seqs methods of the q2-taxa plug-in. The genus-level compositions of the top 11 abundant ASVs were visualized as a bar plot using the q2-taxa plug-in. For the calculation of the beta-diversity index, the Bray-Curtis dissimilarity index was calculated using the q2-diversity plug-in and then used for PCoA plots. Mann-Whitney U test was performed using the “stats.mannwhitneyu” function of the python library “SciPy” (version 1.3.0). Feature selection and individual prediction were performed using SVM-RFE with linear kernel, as described above.

### Data availability.

Spacer sequences are available in the SRA database under accession numbers DRA009650 and DRA010353. Meta 16S rRNA sequences are available in the SRA database under accession number DRA010342.
