# Supplementary material for: Evaluation of CRISPR Diversity in the Human Skin Microbiome for Personal Identification
Source: mSystems. 2021 Feb 2;6(1):e01255-20. doi: 10.1128/mSystems.01255-20 (PMC7857535; doi:10.1128/mSystems.01255-20)
Supplement: FIG S1 [file mSystems.01255-20-sf001.pdf]

A

Sequence description : flag=1 multi=87.9076 len=13578
Length (bp): 13579

AT%: 60.8660431548715

Number of CRISPRs candidates: 1

CRISPRs

Candidate number 1

CRISPR ID : k99_9627_r_1

- Left flanking sequence: (100 bp [start=2497;end=2596], AT%=68): CTTGCAGTCTTACTTCTCTACAAGGGAATGGGAATGTGACGACTAATATGTAAACTTGAATAAGAAAAAAGCTACTTAAAAAATGAGTAGCTTTTAATGT
- CRISPR start position: 2597 ---------- CRISPR end position: 3292 ---------- CRISPR length: 695
- DR consensus: GTTTTGGAACCATTCGAAACAACACAGCTCTAAAAC
- DR length: 36 Number of spacers: 10
- Consensus Repeat ID (Nb in CRISPRdb): Unknown (0)
- CRISPRDirection: ND
- Potential orientation (AT%): +
- Conservation of DRs based on Entropy: 97.5583500726583
- Conservation of Spacers: 0
- Evidence Level: 4

2597	GATTTGGAACTATTCGAAACAACACAGCTCTAAAAC	CCTGTGAACTCAGGAATATCTAAAGTCTCA	2662
2663	GTTTTGGAACCATTCGAAACAACACAGCTCTAAAAC	AATCGTCAAGAAGATATTGCTAAATTATTT	2728
2729	GTTTTGGAACCATTCGAAACAACACAGCTCTAAAAC	TTCACTCTGAATATGTGGTCTATAGACCGT	2794
2795	GTTTTGGAACCATTCGAAACAACACAGCTCTAAAAC	AGTCAGATAGCGAATCTACAGATCCTGTTA	2860
2861	GTTTTGGAACCATTCGAAACAACACAGCTCTAAAAC	ACGGCAATCTTTGAGTGTGAGGTTTCAACC	2926
2927	GTTTTGGAACCATTCGAAACAACACAGCTCTAAAAC	TGACGCGTCGATTTGATCGTCGTGCTTTTA	2992
2993	GTTTTGGAACCATTCGAAACAACACAGCTCTAAAAC	CGGGCGGAGCCCGTGTCTAAAGTTGGTAAA	3058
3059	GTTTTGGAACCATTCGAAACAACACAGCTCTAAAAC	TCTCAACGGTCATATTATCGAGACCTATAT	3124
3125	GTTTTGGAACCATTCGAAACAACACAGCTCTAAAAC	TCAGGCGTTGCATTAACTTCAGTAACTAAT	3190
3191	GTTTTGGAACCATTCGAAACAACACAGCTCTAAAAC	AGAAGCTAAAGCCTCTCGGCTGGCTGGCAA	3256
3257	GTTTTGGAACCATTCGAAACAACACAGCTCTAAAAC		3292

- Right flanking sequence: (100 bp [start=3293;end=3392], AT%=59): CTCGTAGAAAAATTTTTCTCACGAAATTTCGTAATCGCGCCATTCGTCCCAGCCAGACTTCAGCTCGTCAGGTTCCAATTGTTACTCTTATTATACCATA

Cas system(s) and genes

- System number 1: CAS:begin=3383;end=9420:{sequenceID=k99_9627_r} : [cas2_TypeI-II-III (4035,4379,-); cas1_TypeII (4376,5242,-); cas9_TypeII (5242,9420,-); csn2_TypeIIA (3383,4045,-)]
- System number 2: CAS-TypeIIA:begin=3383;end=4045:{sequenceID=k99_9627_r} : [csn2_TypeIIA (3383,4045,-)]
- System number 3: CAS-TypeIIU:begin=3383;end=9420:{sequenceID=k99_9627_r} : [cas9_TypeII (5242,9420,-); cas1_TypeII (4376,5242,-); csn2_TypeIIA (3383,4045,-)]

B

Sequence description : Streptococcus oralis strain FDAARGOS_367 chromosome, complete genome
Length (bp): 2053209

AT%: 59.0601833520114

Number of CRISPRs candidates: 1

CRISPRs

Candidate number 1

CRISPR ID : CP023507_1

- Left flanking sequence: (100 bp [start=2004763;end=2004862], AT%=61): ACCGTACTTTTTATCGCACTAGCCGTTTTCCTTTGCTATAAGGGAATGACCTATGTGTAGGGGGGAATAGTTAAAAAGCTACTCAAAAAATGAGTAGCTT
- CRISPR start position: 2004863 ---------- CRISPR end position: 2005492 ---------- CRISPR length: 629
- DR consensus: GTTTTGGAACCATTCGAAACAACACAGCTCTAAAAC
- DR length: 36 Number of spacers: 9
- Consensus Repeat ID (Nb in CRISPRdb): Unknown (0)
- CRISPRDirection: ND
- Potential orientation (AT%): +
- Conservation of DRs based on Entropy: 98.697234462252
- Conservation of Spacers: 0
- Evidence Level: 4

2004863	TTTTTGGAACCATTCGAAACAACACAGCTCTAAAAC	TGAGTCAAAAGCAACATGACTTTTAGTGGT	2004928
2004929	GTTTTGGAACCATTCGAAACAACACAGCTCTAAAAC	CTACCTCAATCCTTGGATTTAAGCTGTAAA	2004994
2004995	GTTTTGGAACCATTCGAAACAACACAGCTCTAAAAC	ACTTTGACAAGCAATAGCTATCAAGAAAAT	2005060
2005061	GTTTTGGAACCATTCGAAACAACACAGCTCTAAAAC	AGAGCCATCCTTACACTATTCAGAGGCCTT	2005126
2005127	GTTTTGGAACCATTCGAAACAACACAGCTCTAAAAC	TTTTCATCAAGCGCCAATCCATCCAGTAAA	2005192
2005193	GTTTTGGAACCATTCGAAACAACACAGCTCTAAAAC	TTCCTTATCTTGATTATATTATATCACGGT	2005258
2005259	GTTTTGGAACCATTCGAAACAACACAGCTCTAAAAC	AACAATAATGACAGCAGAAAGAGGACACGA	2005324
2005325	GTTTTGGAACCATTCGAAACAACACAGCTCTAAAAC	CCTGCTGAGGCACAGCAATCACTTTCAGAC	2005390
2005391	GTTTTGGAACCATTCGAAACAACACAGCTCTAAAAC	TGTTGTAGCATTTCAGCAAGCGACTGTTCT	2005456
2005457	GTTTTGGAACCATTCGAAACAACACAGCTCTAAAAC		2005492

- Right flanking sequence: (100 bp [start=2005493;end=2005592], AT%=58): CTCGTAGAAAAGTTTTTCTCACGAAATTTCGTAATCGCGCCATTCGTCCCAGCCAGACTTCAGCTCGTTAGGTTCCAATTGTTACCCTTATTATACCATA

Cas system(s) and genes

- System number 1: CAS-TypeIIA:begin=2005583;end=2011620:{sequenceID=CP023507} : [csn2_TypeIIA (2005583,2006245,-); cas2_TypeI-II-III (2006235,2006579,-); cas1_TypeII (2006576,2007442,-); cas9_TypeII (2007442,2011620,-)]

C

Sequence description : flag=0 multi=195.2446 len=7731
Length (bp): 7731

AT%: 68.0119001422843

Number of CRISPRs candidates: 1

CRISPRs

Candidate number 1

CRISPR ID : **k99_91515_1**

- Left flanking sequence: (100 bp [start=6304;end=6403], AT%=72): CCAATGTTATTGAAATATTTGAAAGAATGGTATTGACGAGTTGGATTTAAGATTTTATAATGGTTTTGAGGGAACAAAAATCAAAATTGACGAATTTGAG
- CRISPR start position: 6404 ---------- CRISPR end position: 7690 ---------- CRISPR length: 1286
- DR consensus: GTTTTTGTACTCTCAAGATTTAAGTAACTGTACAAC
- DR length: 36 Number of spacers: 19
- Consensus Repeat ID (Nb in CRISPRdb): Unknown (0)
- CRISPRDirection: ND
- Potential orientation (AT%): .
- Conservation of DRs based on Entropy: 100
- Conservation of Spacers: 0
- Evidence Level: 4

6404	GTTTTTGTACTCTCAAGATTTAAGTAACTGTACAAC	CGCATCCGTTGCATCTGGAACATACTTTTG	6469
6470	GTTTTTGTACTCTCAAGATTTAAGTAACTGTACAAC	TTTTAACCTTCAATTTCAGTTTATGAATCA	6535
6536	GTTTTTGTACTCTCAAGATTTAAGTAACTGTACAAC	CTTTACTCTCTGAGTTCCATTTTTAGAGTT	6601
6602	GTTTTTGTACTCTCAAGATTTAAGTAACTGTACAAC	ATTTAGGTACAGAAAGCGTTATGAATAGCC	6667
6668	GTTTTTGTACTCTCAAGATTTAAGTAACTGTACAAC	CTTCTGCATCGATTACCACAAATAGGACAG	6733
6734	GTTTTTGTACTCTCAAGATTTAAGTAACTGTACAAC	AATCTGATAGGTGAAAGCAATATATCAGCC	6799
6800	GTTTTTGTACTCTCAAGATTTAAGTAACTGTACAAC	TAGTTTCAGCAATCAATGCTGTTATTTAAT	6865
6866	GTTTTTGTACTCTCAAGATTTAAGTAACTGTACAAC	CTGGCTTTATCCATGTTGAAAGCGTTAGC	6930
6931	GTTTTTGTACTCTCAAGATTTAAGTAACTGTACAAC	GTTCATTTAAGCTGATAGTGTATAGACTGG	6996
6997	GTTTTTGTACTCTCAAGATTTAAGTAACTGTACAAC	GCTTTTAATTGTGTATGCAATGTTTGCAA	7061
7062	GTTTTTGTACTCTCAAGATTTAAGTAACTGTACAAC	GCAGTATTTGATATTCATCATTATTAAAAT	7127
7128	GTTTTTGTACTCTCAAGATTTAAGTAACTGTACAAC	AGAAGATAGCTCAACTCGTTACCAAAATCG	7193
7194	GTTTTTGTACTCTCAAGATTTAAGTAACTGTACAAC	AGAACCATTGAAACAGCCTATAAAAACATT	7259
7260	GTTTTTGTACTCTCAAGATTTAAGTAACTGTACAAC	CTGATGTAAACATTTTATCAATGAACTCGT	7325
7326	GTTTTTGTACTCTCAAGATTTAAGTAACTGTACAAC	TGGAAAATAAAAAAGCGCCTTCCCGATTG	7390
7391	GTTTTTGTACTCTCAAGATTTAAGTAACTGTACAAC	CGACTTTTAATATCCTTGATTAGTTCTCTT	7456
7457	GTTTTTGTACTCTCAAGATTTAAGTAACTGTACAAC	TCTACCAATGAAATGTGACCCGAGCATTGT	7522
7523	GTTTTTGTACTCTCAAGATTTAAGTAACTGTACAAC	TACCAAGGACGATTGACGAAAATCACAAGT	7588
7589	GTTTTTGTACTCTCAAGATTTAAGTAACTGTACAAC	CATGTGATAAGCTGTTTTTACAAGTCTTTT	7654
7655	GTTTTTGTACTCTCAAGATTTAAGTAACTGTACAAC		7690

- Right flanking sequence: (100 bp [start=7691;end=7790], AT%=0): UNKNOWN

Cas system(s) and genes

- System number 1: CAS:begin=474;end=5293:{sequenceID=k99_91515} : [cas2_TypeI-II-III (4970,5293,+); cas1_TypeII (3853,4965,+); cas9_TypeII (474,3878,+)]
- System number 2: CAS-TypeIIU:begin=474;end=4965:{sequenceID=k99_91515} : [cas9_TypeII (474,3878,+); cas1_TypeII (3853,4965,+)]

D

Sequence description : Streptococcus mitis strain S022-V3-A4 chromosome, complete genome
Length (bp): 27691

AT%: 61.4604022967751

Number of CRISPRs candidates: 1

CRISPRs

Candidate number 1

CRISPR ID : CP047883_1

- Left flanking sequence: (100 bp [start=8054;end=8153], AT%=74): TTTATATAAGTTAGACAATTGCTAATATTATCTAAATATACAGTTACTTAAATCTTGAGAGTACAAAAACCGTCACGGGATTTCTATATCTGGATTTTAA
- CRISPR start position: 8154 --------- CRISPR end position: 11289 --------- CRISPR length: 3135
- DR consensus: GTTGTACAGTTACTTAAATCTTGAGAGTACAAAAAC
- DR length: 36 Number of spacers: 47
- Consensus Repeat ID (Nb in CRISPRdb): Unknown (0)
- CRISPRDirection: ND
- Potential orientation (AT%): .
- Conservation of DRs based on Entropy: 100
- Conservation of Spacers: 0
- Evidence Level: 4

8154	GTTGTACAGTTACTTAAATCTTGAGAGTACAAAAAC	AGTAGTTGCTTACAACGTTTACCGTTATTA	8219
8220	GTTGTACAGTTACTTAAATCTTGAGAGTACAAAAAC	TGTCACTTATCTCCTCGCAAACCCTTGATA	8285
8286	GTTGTACAGTTACTTAAATCTTGAGAGTACAAAAAC	TATGTTCTTGAACTTGTTTTGCAAGCTCTG	8351
8352	GTTGTACAGTTACTTAAATCTTGAGAGTACAAAAAC	AAGTGGGCCTGGCAGGTAGGCTTGTGACCG	8417
8418	GTTGTACAGTTACTTAAATCTTGAGAGTACAAAAAC	AGTCTACCCAAGTAGATAGGCTCTTGAGAT	8483
8484	GTTGTACAGTTACTTAAATCTTGAGAGTACAAAAAC	ACTTGGGTTGACTTGAAAGGGTCAGAGTAT	8549
8550	GTTGTACAGTTACTTAAATCTTGAGAGTACAAAAAC	CTACTTAATAAAGATGTAAAAACTGTTCGA	8615
8616	GTTGTACAGTTACTTAAATCTTGAGAGTACAAAAAC	CAACCAGATTTGCTTCCCACGCTTTGCGGG	8681
8682	GTTGTACAGTTACTTAAATCTTGAGAGTACAAAAAC	AGTCAGTGTAAGAGACTTCAGCAAGTGATT	8747
8748	GTTGTACAGTTACTTAAATCTTGAGAGTACAAAAAC	TCTTTGACGTGCTTTACATCAATGAAGACG	8813
8814	GTTGTACAGTTACTTAAATCTTGAGAGTACAAAAAC	CATCTTCAAAGCCACCCATCAAAGCGGTAT	8879
8880	GTTGTACAGTTACTTAAATCTTGAGAGTACAAAAAC	CAAATGCTGTTGCCAAAGGAGAAGCATCAA	8945
8946	GTTGTACAGTTACTTAAATCTTGAGAGTACAAAAAC	CTCGAATTTTAGCCGTAATAGAGTTGTCTG	9011
9012	GTTGTACAGTTACTTAAATCTTGAGAGTACAAAAAC	ACAAATCAAAGTTACCTTTCATTTGCAATT	9077
9078	GTTGTACAGTTACTTAAATCTTGAGAGTACAAAAAC	CCGCTCTAATATCAACTAAGGGAGTATAG	9142
9143	GTTGTACAGTTACTTAAATCTTGAGAGTACAAAAAC	CGCTGCGACAAAGTACGGGACAGTTTATGT	9208
9209	GTTGTACAGTTACTTAAATCTTGAGAGTACAAAAAC	CTTTTTTTTGCTATACTAGTATCGCTCTCG	9274
9275	GTTGTACAGTTACTTAAATCTTGAGAGTACAAAAAC	AAACAGCGCTTTTAAACGTATTATCAATTT	9340
9341	GTTGTACAGTTACTTAAATCTTGAGAGTACAAAAAC	TATATCCGCTTTAAAATCGTGGGCGTTTAT	9406
9407	GTTGTACAGTTACTTAAATCTTGAGAGTACAAAAAC	CAGCAATGCTATCATCACTTGTTGCTTTAA	9472
9473	GTTGTACAGTTACTTAAATCTTGAGAGTACAAAAAC	AAACAGCGCTTTTAAACGTATTATCAATTT	9538
9539	GTTGTACAGTTACTTAAATCTTGAGAGTACAAAAAC	TATATCCGCTTTAAAATCGTGGGCGTTTAT	9604
9605	GTTGTACAGTTACTTAAATCTTGAGAGTACAAAAAC	CAGCAATGCTATCATCACTTGTTGCTTTAA	9670
9671	GTTGTACAGTTACTTAAATCTTGAGAGTACAAAAAC	ACATTACAACACGATTATAGACCAGACCTA	9736
9737	GTTGTACAGTTACTTAAATCTTGAGAGTACAAAAAC	CCCTTCAACATTCCAGTTTGGGGTAAGTAT	9802
9803	GTTGTACAGTTACTTAAATCTTGAGAGTACAAAAAC	AGGTTTGTAACCCTTGATTTTGTCTTTCAT	9868
9869	GTTGTACAGTTACTTAAATCTTGAGAGTACAAAAAC	CAATAGTCATAATGATCTCCATGTCGTTCT	9934
9935	GTTGTACAGTTACTTAAATCTTGAGAGTACAAAAAC	CTTTGGGCTATGACTTCGTTCAATGGTCAA	10000
10001	GTTGTACAGTTACTTAAATCTTGAGAGTACAAAAAC	CAACTATTAAAGATAAAAAGCTTGTTGGAA	10066
10067	GTTGTACAGTTACTTAAATCTTGAGAGTACAAAAAC	AAGATGATGAAGAATTTAACGGTTACTTG	10131
10132	GTTGTACAGTTACTTAAATCTTGAGAGTACAAAAAC	ATGGCACATCGAATTTGGATAGTGAGAAAT	10197
10198	GTTGTACAGTTACTTAAATCTTGAGAGTACAAAAAC	AGAGGGAATACGTTATACATTCTTGGCTTG	10263
10264	GTTGTACAGTTACTTAAATCTTGAGAGTACAAAAAC	TTAAATCAGATAATTCACAAAAAGCATCCA	10329
10330	GTTGTACAGTTACTTAAATCTTGAGAGTACAAAAAC	CGTATATGTAATTTCTTTAATCTGGTCGTA	10395
10396	GTTGTACAGTTACTTAAATCTTGAGAGTACAAAAAC	TTCTGAACAATGATATAGAATGTACTTATT	10461
10462	GTTGTACAGTTACTTAAATCTTGAGAGTACAAAAAC	AGCGCATATAAACTGATTCAAAAGCACTCA	10527
10528	GTTGTACAGTTACTTAAATCTTGAGAGTACAAAAAC	AAGAGAATCGGGACATGCTGAAACAGAGCG	10593
10594	GTTGTACAGTTACTTAAATCTTGAGAGTACAAAAAC	AGATAATCTTCGTATGGATTTTGCTGGCAT	10659
10660	GTTGTACAGTTACTTAAATCTTGAGAGTACAAAAAC	TTTTTATGCTACTGTCACTAAGCCGTCTGG	10725
10726	GTTGTACAGTTACTTAAATCTTGAGAGTACAAAAAC	CAAAAGGGAAGGCGCTTTTTATTTTCCAAA	10791
10792	GTTGTACAGTTACTTAAATCTTGAGAGTACAAAAAC	TGCAGATAACGCTGAAATGTATGGTGACCA	10857
10858	GTTGTACAGTTACTTAAATCTTGAGAGTACAAAAAC	AGCGAGTGAAATTTTCCAATAAACGTCAGT	10923
10924	GTTGTACAGTTACTTAAATCTTGAGAGTACAAAAAC	TAACAGGGAAATCGGTTTGGAGACTTTCGC	10989
10990	GTTGTACAGTTACTTAAATCTTGAGAGTACAAAAAC	AGGCGTTGTTTTCTGTTGTGATGCGGTTGT	11055
11056	GTTGTACAGTTACTTAAATCTTGAGAGTACAAAAAC	AAGGCTTGTCTAGTGCTGGCAAGATGGTTA	11121
11122	GTTGTACAGTTACTTAAATCTTGAGAGTACAAAAAC	TATAAATAACTCGTCAATATCATTAGGCAA	11187
11188	GTTGTACAGTTACTTAAATCTTGAGAGTACAAAAAC	AACTTTGCACGCAGTCAAGGGAAATCACTT	11253
11254	GTTGTACAGTTACTTAAATCTTGAGAGTACAAAAAC		11289

- Right flanking sequence: (100 bp [start=11290;end=11389], AT%=0): UNKNOWN

Cas system(s) and genes

- System number 1: CAS-TypeIIU:begin=4337;end=17219:{sequenceID=CP047883} : [cas9_TypeII (13815,17219,-); cas1_TypeII (12728,13840,-); cas3_TypeI (4337,5419,+)]
- System number 2: CAS:begin=4337;end=17219:{sequenceID=CP047883} : [cas2_TypeI-II-III (12400,12723,-); cas1_TypeII (12728,13840,-); cas3_TypeI (4337,5419,+); cas3_TypeI (4337,5419,+); cas9_TypeII (13815,17219,-)]
