# Supplementary figures and images for: Evaluation of CRISPR Diversity in the Human Skin Microbiome for Personal Identification
Source: mSystems. 2021 Feb 2;6(1):e01255-20. doi: 10.1128/mSystems.01255-20 (PMC7857535; doi:10.1128/mSystems.01255-20)

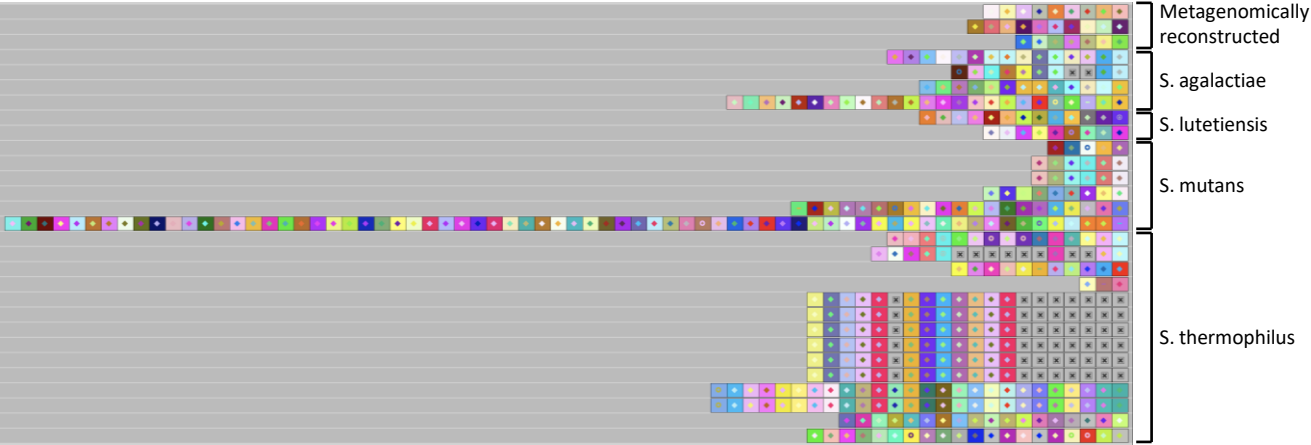

Supplement: FIG S2 [file mSystems.01255-20-sf002.pdf]

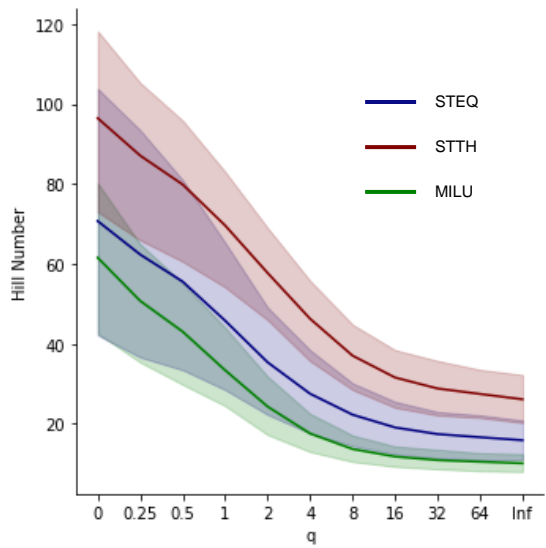

Supplement: FIG S3 [file mSystems.01255-20-sf003.pdf]

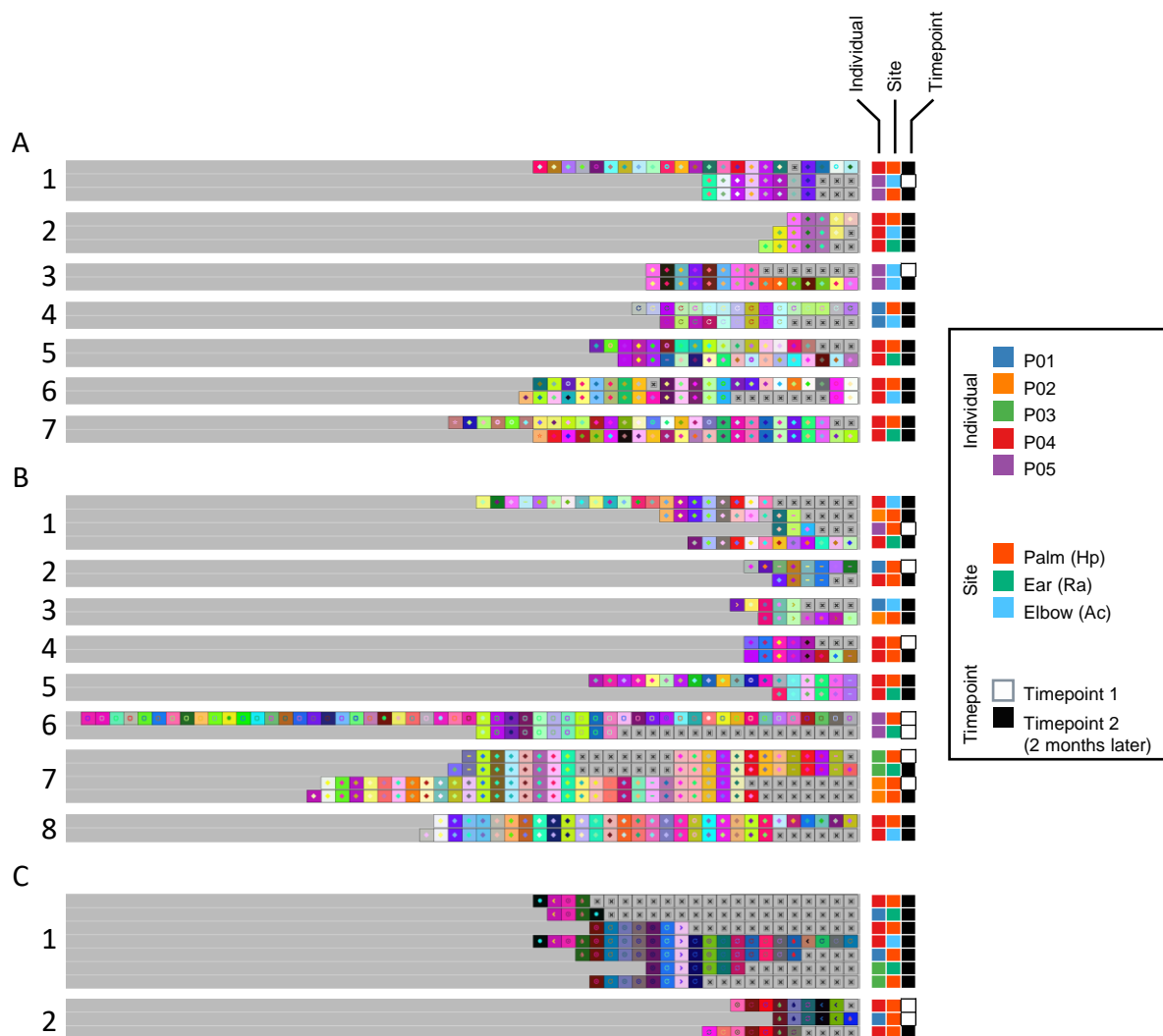

Supplement: FIG S4 [file mSystems.01255-20-sf004.pdf]

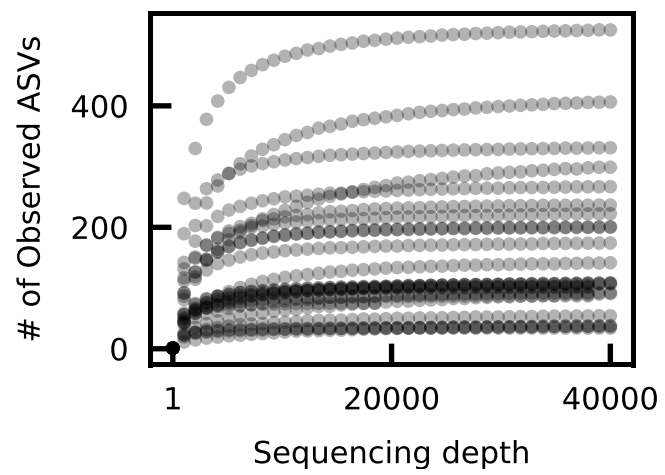

Supplement: FIG S5 [file mSystems.01255-20-sf005.pdf]
