# Supplementary material for: Evaluation of CRISPR Diversity in the Human Skin Microbiome for Personal Identification
Source: mSystems. 2021 Feb 2;6(1):e01255-20. doi: 10.1128/mSystems.01255-20 (PMC7857535; doi:10.1128/mSystems.01255-20)
Supplement: FIG S6 [file mSystems.01255-20-sf006.pdf]

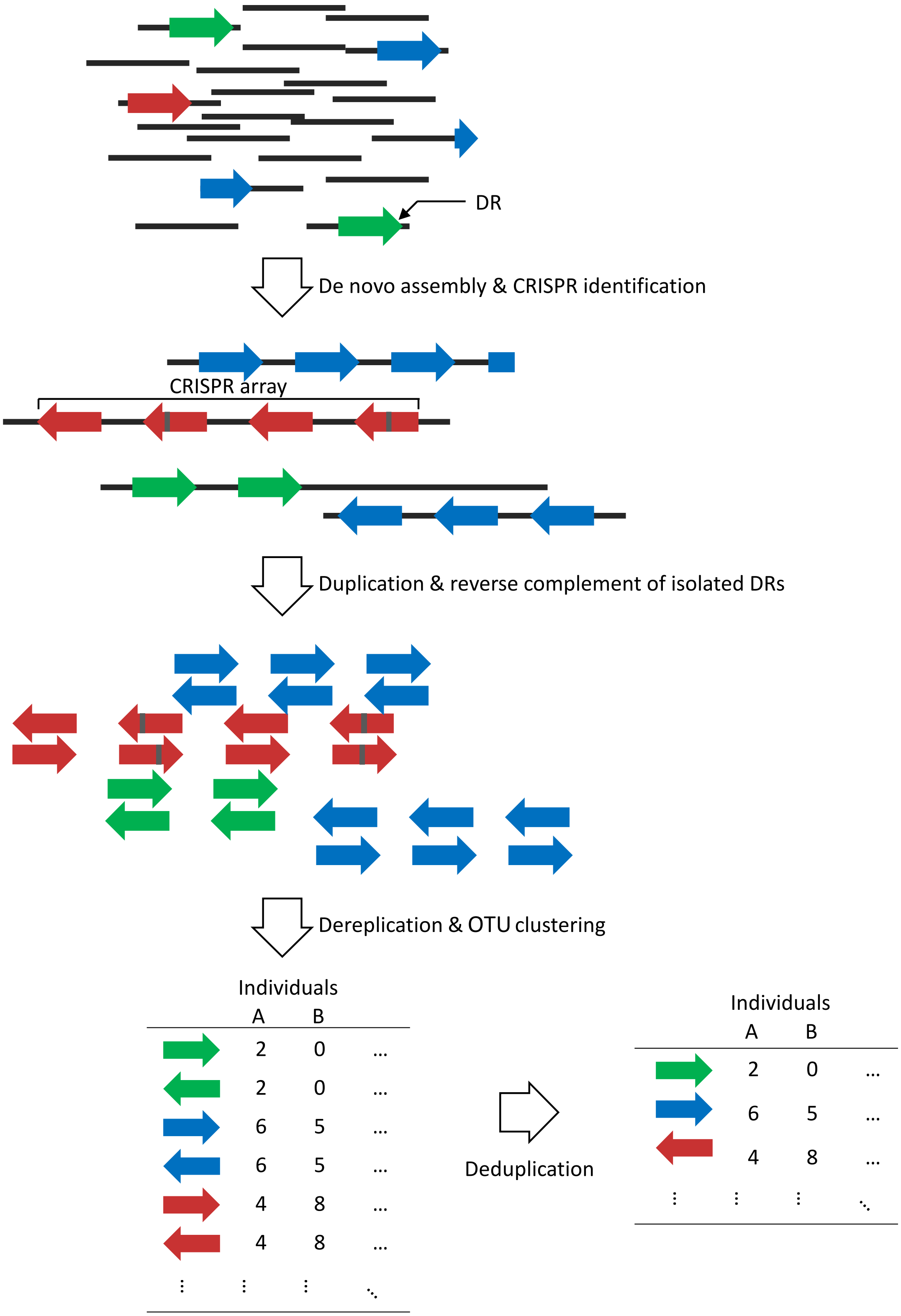

DR
De novo assembly & CRISPR identification
CRISPR array
Duplication & reverse complement of isolated DRs
Dereplication & OTU clustering
Individuals
A B
2 0 ...
2 0 ...
6 5 ...
6 5 ...
4 8 ...
4 8 ...
Deduplication
Individuals
A B
2 0 ...
6 5 ...
4 8 ...
